# Cigarette smoke-induced impairment of autophagy in macrophages increases galectin-8 and inflammation

**DOI:** 10.1038/s41598-020-79848-0

**Published:** 2021-01-11

**Authors:** Yuta Kono, Thomas Colley, Masako To, Andriana I. Papaioannou, Nicolas Mercado, Jonathan R. Baker, Yasuo To, Shinji Abe, Kosuke Haruki, Kazuhiro Ito, Peter J. Barnes

**Affiliations:** 1 National Heart and Lung Institute, Imperial College London, London, UK; 2 Deparment of Allergy and Respiratory Medicine, The Fraternity Memorial Hospital, Tokyo, Japan; 3 Dapartment of Laboratory Medicine, Dokkyo Medical University Saitama Medical Centre, Saitama, Japan; 4 3rd Respiratory Medicine Department, Sismanogleio Hospital, Marousi, Athens, Greece; 5 Department of Respiratory Medicine, Tokyo Medical University Hospital, Tokyo, Japan

**Keywords:** Macroautophagy, Chronic obstructive pulmonary disease

## Abstract

Cigarette smoke impairs autophagy, an intracellular protein degradation system, but the consequences of this defect have not been fully elucidated, especially in macrophages. Dysfunctional alveolar macrophages play an important role in chronic obstructive pulmonary disease (COPD). Here we show that galectin-8, a danger receptor that identifies damaged intracellular host vesicles and initiates autophagosome engulfment, is elevated due to activation of autophagy by cigarette smoke extract (CSE) in macrophages. CSE impaired autophagic flux in PMA-differentiated U937 macrophage-like cells, resulting in intracellular accumulation of galectin-8 and the autophagic adaptor protein NDP52. COPD patients showed elevated levels of galectin-8 and NDP52 in the lung homogenates with significant increase in the serum galectin-8 levels in patients with frequent acute exacerbations. Soluble galectin-8 induced interleukin (IL)-6 release in bronchial epithelial cells via PI3Kα signalling. Thus, increased galectin-8 due to CSE-induced impaired autophagy may be involved in the pathogenesis of COPD and may be a biomarker of this disease.

## Introduction

Macroautophagy (hereafter termed autophagy) is an intracellular degradation system which delivers dysfunctional cytoplasmic constituents via autophagosomes to lysosomes, resulting in formation of an autolysosome or autophagolysosome, depending on the cargo^1,2^. In selective autophagy, misfolded and damaged proteins, non-functional organelles or intracellular pathogens are tagged with ubiquitin chains by E3 ubiquitin ligases and, depending on the chain type, undergo targeted degradation^3^. Autophagic selectivity is mediated by autophagy adaptor proteins which target specific moieties and interact with autophagy proteins bound to autophagosomal membranes, including microtubule-associated proteins 1A/1B light chain 3B (LC3). Intracellular bacteria are targeted by adaptor proteins including SQSTM1/p62 (p62), nuclear dot protein 52 (NDP52), and optineurin, triggering amino acid starvation, mammalian target of rapamycin (mTOR) deactivation and autophagy induction^4^*.* Autophagy exerts homeostatic functions to maintain intracellular environments and is essential for cell survival. Thus, dysregulation of autophagy has been shown to contribute towards the pathogenesis of many diseases, including Parkinson’s disease, lysosomal storage disorders, cancer and Crohn’s disease^5^.

Chronic obstructive pulmonary disease (COPD) is the 3rd leading cause of death in the world, and a chronic respiratory disease characterised by a persistent airway inflammatory response to noxious gas and particles, such as cigarette smoke and biomass smoke, leading to progressive airflow limitation, small airway fibrosis and emphysema^6^. Several mechanisms are involved in the development of COPD, such as oxidative stress, corticosteroid-resistant inflammation, activation of the innate immune response^6^, and increased cellular senescence^7,8^. Within the last decade, several studies have linked cellular defects induced by cigarette smoke with the pathogenesis of COPD. Autophagosome number and LC3 expression are increased in lung tissue from COPD patients, while LC3B^−/−^ mice appear to be protected from many pathological features associated with cigarette smoking, including decreased lung airspace, and amplified apoptosis^9,10^. Similarly, in bronchial epithelial cells cigarette smoke extract (CSE) increases LC3B-II expression and induces autophagy while knock-down of autophagy proteins LC3B and Beclin-1 protect against CSE-induced apoptosis^11^. Despite initial evidence suggesting that increased autophagosome number were associated with excessive autophagy activation, more recent data suggests that autophagosome accumulation is the result of defective autophagosome maturation. Assessment of autophagic flux in bronchial epithelial cells indicates that autophagy is impaired by CSE and associated with aggresome formation^12^. Moreover, we have recently shown that defective autophagosome maturation is the result of bicaudal D homolog 1 (BICD1) accumulation which impairs autophagosome maturation^13^. The interplay between cigarette smoking, autophagy and macrophage function has also been reported, with alveolar macrophages from smokers showing accumulation of autophagosomes and protein aggregates due to defective autophagic flux^14^.

Galectin-8 is a danger receptor that identifies damaged intracellular host vesicles and initiates selective autophagosome engulfment and degradation via interaction with the autophagic adaptor protein NDP52^15,16^*.* This process prevents leakage or escape of potentially harmful cargo, including protein aggregates, bacteria and virions, from damaged vesicles and facilitates secondary containment and destruction^15,17–19^. Galectin-8 also modulates a number of known inflammatory pathways, modulating cell adhesion, promoting apoptosis and inducing senescence^20–22^. In this study, we have investigated the role of CSE on galectin-8/NDP52 signalling in macrophages and its implication.

## Results

### Cigarette smoke exposure impairs autophagic flux in macrophages

Firstly, the impact of CSE exposure on the autophagic markers p62 and LC3, which were quantified by Western blot, were investigated in macrophages using phorbol 12-myristate 13-acetate (PMA)-dependent differentiated macrophage-like human U937 cells.

**Figure 1:**
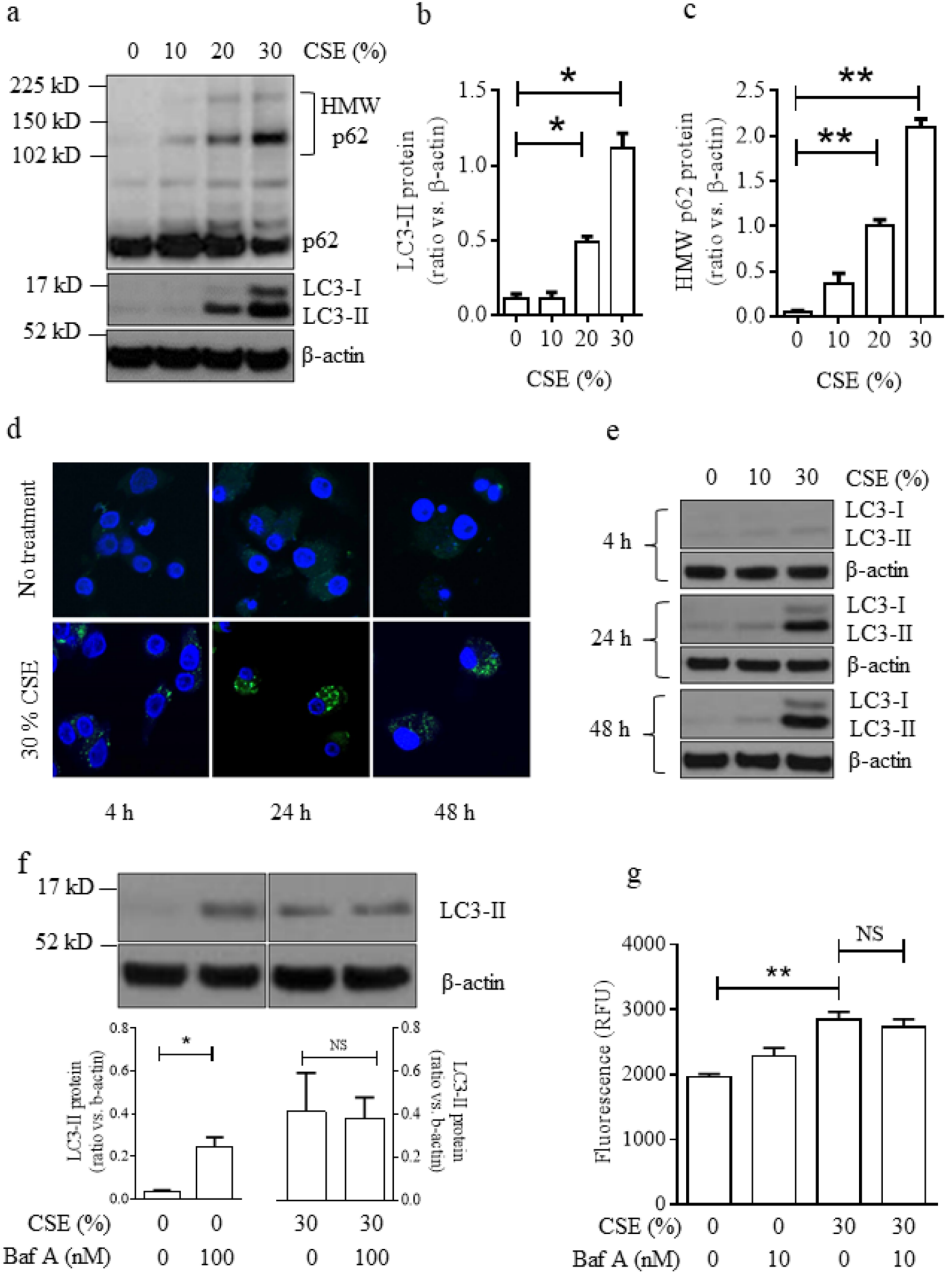
Cigarette smoke exposure impairs autophagosome maturation in PMA-differentiated U937 cells. (**a**) Protein expression of LC3-I/II and HMW-p62 and the band densities of Western blotting analysis of LC3-II (**b**) and HMW-p62 (**c**) n = 3, (**d**) confocal imaging (× 63) of GFP-LC3 transfected, CSE treated cells; representative images from 3 independent experiments; Blue = DAPI, Green = GFP-LC3, (**e**) Time course of LC3I/II expression in CSE exposed cells; n = 3, (**f**) Effects of Baf A on CSE-induced LC3-II expression as LC3 turnover assay in 5 min exposure (left panel) and 30 s exposure (right panel) ; n = 3, (**g**) Long-lived protein degradation in cells exposed to CSE (n = 3). *P < 0.05, **P < 0.01. *CSE* cigarette smoke extract, *NS* not significant, *RFU* relative fluorescent units, *Baf A* bafilomycin A.

The conversion of LC3-I to LC3-II via conjugation with phosphatidylethanolamine signals the formation of autophagosomes and is reliably correlated with autophagosome number^23^. CSE exposure for 24 h resulted in the concentration-dependent accumulation of LC3-I, LC3-II and total LC3 (Fig. 1a,b). In addition, significant autophagosome accumulation in response to CSE treatment for 24 h was observed directly by fluorescence microscopy in these cells transfected with GFP-LC3 (Fig. 1d). LC3-II expression was also detected at 4 h, 24 h and 48 h (with a wash at 24 h followed by the addition of fresh media, to remove CSE in order to minimise reduction of cell viability). It was observed that treatment with 30% CSE significantly increased LC3-II in a time-dependent manner (Fig. 1e, supplementary Fig. S1), and LC3-II concentrations did not recede even after the removal of CSE, suggesting that impaired autophagosome clearance was sustained as shown before^13^. This finding was confirmed visually by monitoring GFP-LC3 punctae accumulation by fluorescence microscopy (Fig. 1d). In contrast, although treatment with 10% CSE slightly increased LC3-II at 24 h, removal of CSE followed by 24 h incubation in the absence of CSE resulted in LC3-II levels decreasing to basal levels by 48 h, suggesting that a low concentration of CSE-induced autophagy, but the effect was only transient (Fig. 1e, supplementary Fig. S1). The cell viability of macrophages treated with CSE are shown in Supplementary Fig. S2.

In addition, accumulation of high molecular weight (HMW)-p62, which is potentially a marker of defective autophagic flux within a cell^13,14^, was observed in cells exposed to CSE for 24 h in a concentration dependent manner (Fig. 1a,c). Furthermore, it was observed that HMW-ubiquitin conjugates were increased in these cells exposed to CSE (Supplementary Fig. S3). In addition, CSE increased expression of Rab7 in macrophages, suggesting altered degradation of late stage endosomes and impaired autophagosome maturation^24^ (Supplementary Fig. S4).

Accumulation of autophagosomes may indicate the induction of autophagy or conversely it could indicate inhibition of autophagosome maturation and lysis^23^. Therefore, the LC3 turnover assay was used to clarify the mechanism responsible for accumulation of autophagosomes in response to CSE. Macrophages were treated with CSE for 24 h, and the rate of autophagic flux (autophagosome maturation and lysis) was evaluated using bafilomycin A (Baf A), a lysosomotropic reagent that raises the pH of the lysosome and inhibits fusion between the autophagosome and lysosome. It was observed that LC3-II was significantly increased by treatment with Baf A for 2 h and by treatment with 30% CSE, whilst cell viability was not significantly affected (Fig. 1f and Supplementary Fig S5). However, the addition of Baf A for 2 h to the cells pre-treated with 30% CSE for 24 h had no further impact on LC3-II levels, indicating that 30% CSE inhibited autophagic flux (Fig. 1f, right panel). To confirm these findings an alternative measurement of autophagic flux was used. Short-lived proteins are mainly degraded by the proteasome, whereas long-lived protein macromolecules are degraded predominantly by the autophagic machinery^23^. It was observed that the quantity of fluorescently labelled long-lived proteins within the cells was increased by CSE exposure (Fig. 1g). Similarly to the LC3 turnover assay, the addition of Baf A to the cells treated with 30% CSE for 24 h had no further impact on the quantity of long-lived proteins, indicating that autophagic flux was impaired by 30% CSE.

### Cigarette smoke disrupts the galectin-8/NDP52 pathway

**Figure 2:**
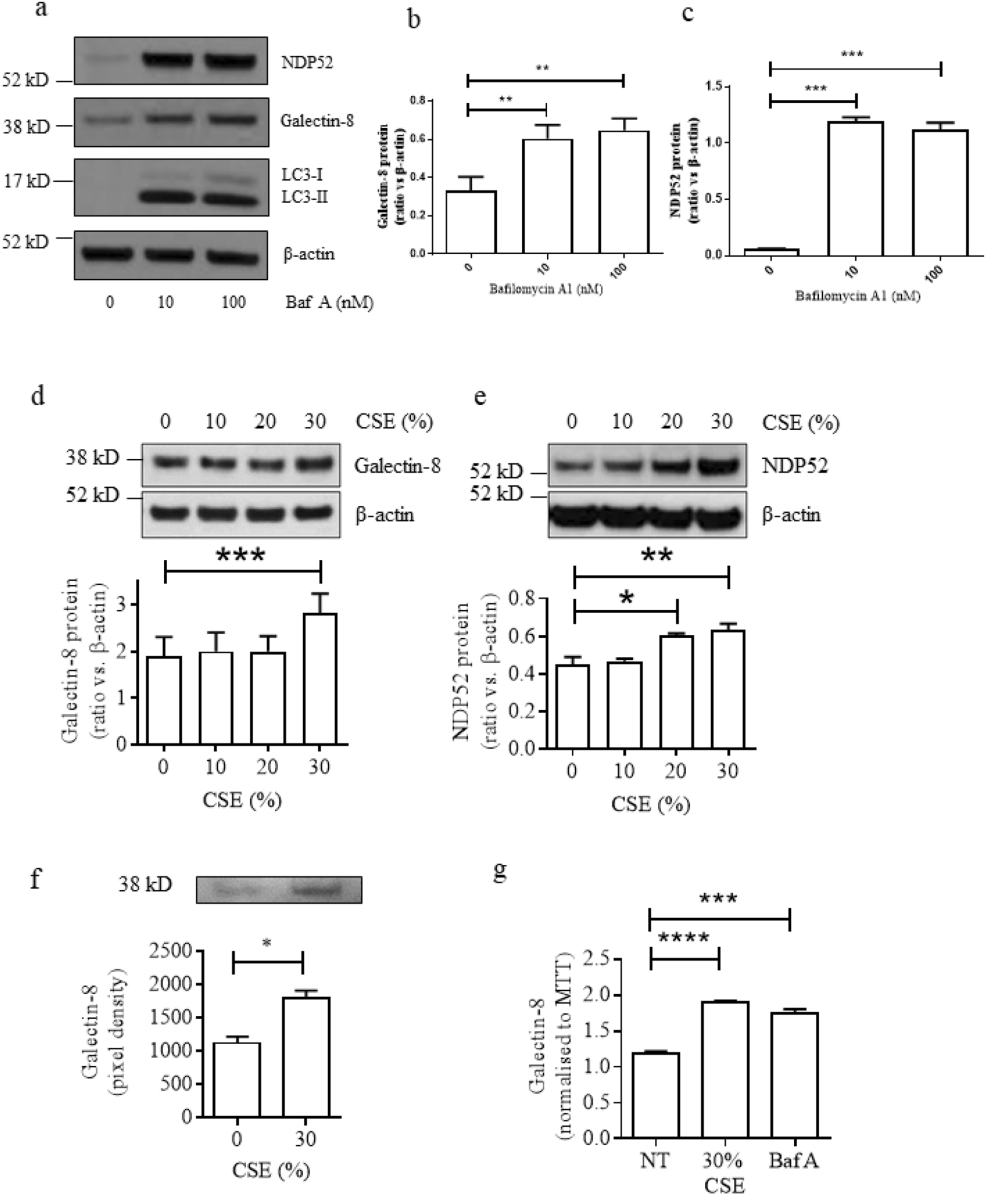
Defective autophagy results in galectin-8 and NDP52 protein accumulation in PMA-differentiated U937 cells. (**a**) NDP52, galectin-8 and LC3 protein expression in cells treated with bafilomycin A; representative images from 3 independent experiments, and the band densities of Galectin-8 (**b**) and NDP52 (**c**) by western blotting analysis, (**d**,**e**) CSE-induced cellular accumulation of galectin-8 (d) and NDP52 (e); n = 3, (**f**) Western blotting analysis and (**g**) ELISA analysis of secreted galectin-8 protein in supernatant from cells exposed to CSE or Baf A (n = 3). *P < 0.05, **P < 0.01, ***P < 0.001, ****P < 0.0001. *Baf A* bafilomycin A, *CSE* cigarette smoke extract, *NT* no treatment.

Next, we evaluated the impact of defective autophagy on autophagy receptor NDP52 and galectin-8 which is a danger receptor recruiting NDP52. Autophagy inhibition by Baf A for 24 h caused accumulation of galectin-8 and NDP52 in PMA-differentiated U937 cells (Fig. 2a–c). In addition, CSE exposure for 24 h produced significant concentration-dependent increases in the levels of galectin-8 and NDP52 (Fig. 2d,e) and secretion of galectin-8 was also increased (Fig. 2f,g) although mRNA levels of galectin-8 and NDP52 were not altered significantly (Supplementary Fig. S6).

### Extracellular release of galectin-8 was NDP52-dependent

**Figure 3:**
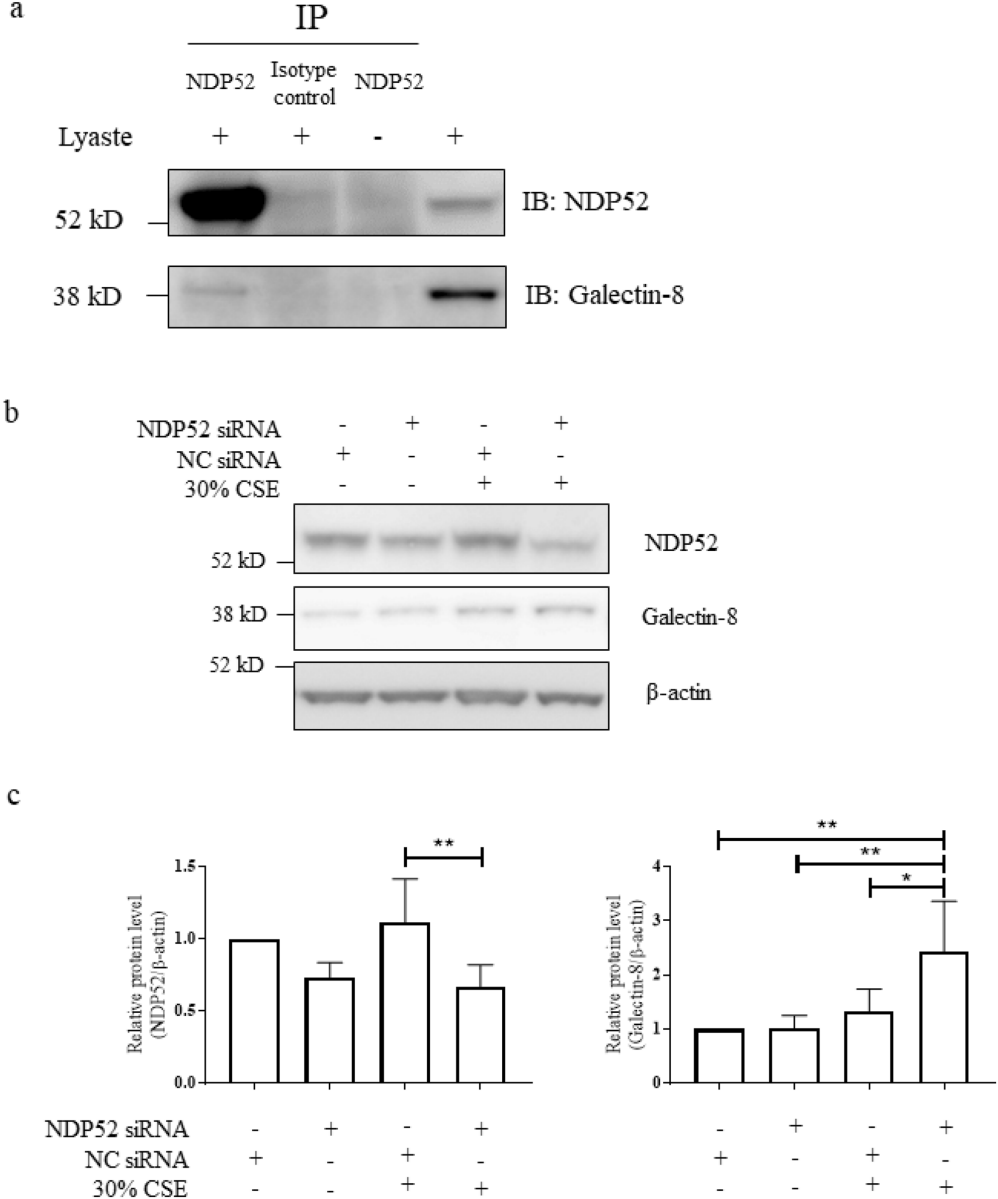
Association of galectin-8 and NDP52. (**a**) Galectin-8 and NDP52 were co-immunoprecipitated using an anti-NDP52 antibody in whole cell lysate of PMA-differentiated U937 cells. (**b**,**c**) Effects of NDP52 knockdown on galectin-8 expression in the presence or absence of 30% CSE (n = 5). *P < 0.05, **P < 0.01. *CSE* cigarette smoke extract, *NC* negative control.

Galectin-8 binding to damaged vesicles triggers the recruitment of NDP52, which in turn interacts with LC3 via a non-canonical ATG8/LC3-interacting region (LIR) motif, leading to autophagy-mediated clearance of the damaged vesicles^15,25^. The interaction between galectin-8 and NDP52 under basal conditions was confirmed in PMA-differentiated U937 cells by co-immunoprecipitation (Fig. 3a). Furthermore, it was observed that knock-down of NDP52 expression combined with exposure to 30% CSE resulted in increased protein levels of galectin-8, when compared to 30% CSE alone (Fig. 3b,c). These data suggest that CSE-induced defective autophagy increases the release of galectin-8 into the extracellular environment through an NDP52-mediated pathway.

### Galectin-8 induces inflammation in bronchial epithelial cells in a PI3Kα-dependent manner

**Figure 4:**
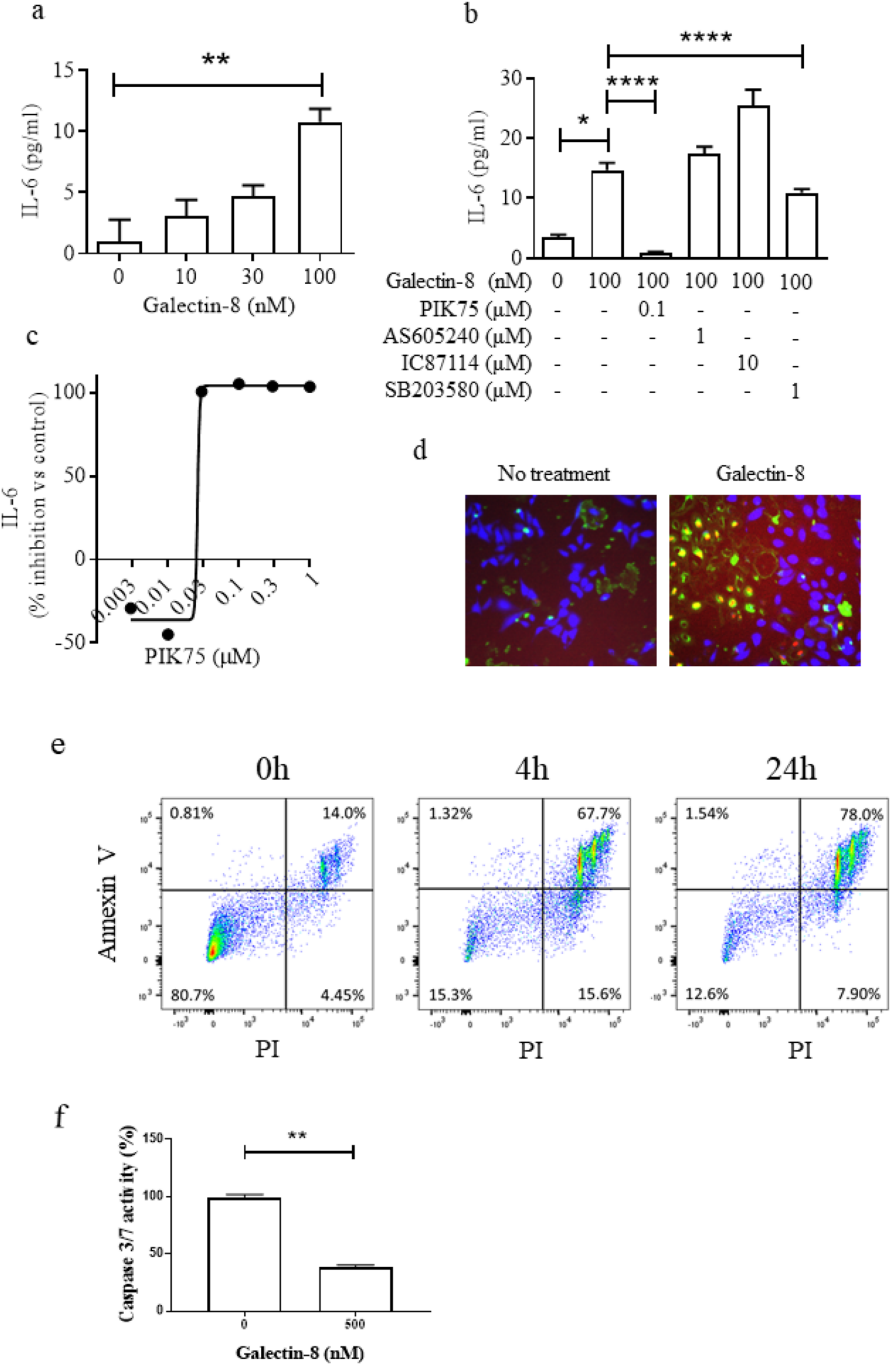
Soluble galectin-8 induces inflammation and apoptosis in BEAS-2B cells. (**a**) Recombinant galectin-8 induced IL-6 protein expression in supernatant from BEAS-2B cells, quantified by ELISA (n = 3), (**b**) Inhibition of galectin-8-induced IL-6 release from BEAS-2B cells by PI3K isoform and p38 MAPK inhibitor; (n = 3), (**c**) Inhibition of galectin-8-induced IL-6 release from BEAS-2B cells by the pan-PI3K inhibitor PIK75 (n = 3), (**d**) Detection of apoptosis in BEAS-2B cells treated with 500 nM recombinant human galectin-8; representative images from 3 independent experiments; Blue = live cells, Green^+^/Red^−^ = early apoptotic cells, Green^+^/Red^+^ = late apoptotic cells or non-apoptotic cells. (**e**) Flowcytometry analysis for apoptosis in BEAS-2B cells treated with 500 nM recombinant human galectin-8. (**f**) Caspase 3/7 activity of BEAS-2B cells treated with 500 nM recombinant human galectin-8. (n = 3). * *P* < 0.05, **P < 0.01, ****P < 0.0001.

To understand the consequence of secreted galectin-8, the effects of recombinant galectin-8 on IL-6 release was investigated in an immortalised human bronchial epithelial cell line (BEAS-2B). It was found that exposure of bronchial epithelial cells to low concentrations of galectin-8 induced expression of IL-6 in a concentration-dependent manner (Fig. 4a), which peaked at 24 h (Supplementary Fig. S7). As shown in Fig. 4b, the galectin-8 dependent IL-6 production was inhibited by the PI3Kα inhibitor PIK75 (IC_50_ = 0.026 μM ± 0.00033, Fig. 4b,c) and, to a lesser extent, the p38 MAPK inhibitor SB203580 (Fig. 4b), but not by PI3Kγ and PI3Kδ inhibitors, while neither galectin-8 nor the inhibitors themselves significantly affected cell viability (Supplementary Fig. S8). In addition, exposure to high concentrations of galectin-8 (500 nM) for 24 h resulted in significantly increased late apoptosis or non-apoptotic cell death (45.0% ± 6.95 positive cells; *P* < 0.05) in BEAS-2B cells when compared to non-treated control cells (12.9% ± 4.10) (Fig. 4d). In order to identify the type of cell death, annexin V-pacific blue/PI staining and flow cytometry were performed. 4 h exposure to high concentration of galectin-8 (500 nM) induced non-apoptotic cell death (annexin V-pacific blue^−^/PI^+^ and annexin V-pacific blue^+^/PI^+^), while early apoptosis (annexin V-pacific blue^+^/PI^−^) mostly was not induced (Fig. 4e). Furthermore, the activities of caspase 3/7 were evaluated. The exposure to high concentration of galectin-8 (500 nM) for 24 h significantly reduced the activities of caspase 3/7, indicating that the cell death caused by galectin-8 was non-apoptotic cell death (Fig. 4f).

### Galectin-8 and NDP52 increased in COPD lungs

**Figure 5:**
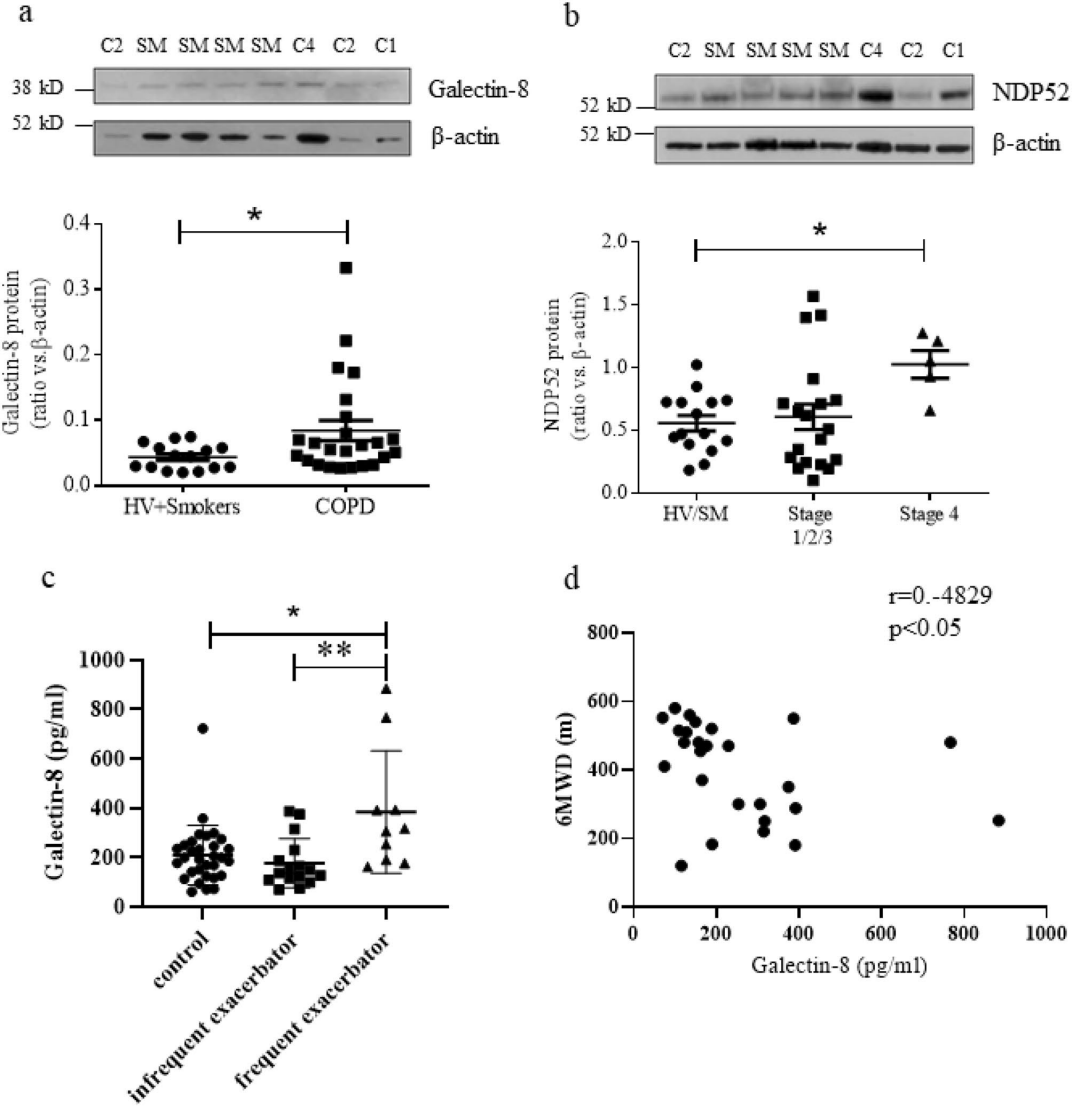
Expression of galectin-8 and NDP52 is increased in COPD. (**a**) Galectin-8 and (**b**) NDP52 protein expression in lung homogenates from healthy volunteers (HV, n = 4), smokers with normal lung function (SM, n = 11), COPD GOLD stage 1 (n = 8), COPD GOLD stage 2 (n = 7), COPD GOLD stage 3 (n = 4) and COPD GOLD stage 4 (n = 5), (**c**) serum galectin-8 of HV + SM (n = 25), COPD patients with infrequent exacerbations (n = 23) and COPD patients with frequent exacerbations (n = 10) as determined by ELISA. (**d**) Correlation between serum galectin-8 and 6-min walking distance (6MWD) in COPD patients (n = 33). *P < 0.05, **P < 0.01. HV = healthy volunteer, SM = smoker, C1/2/4 = COPD GOLD stage 1/2/4.

The protein expressions of galectin-8 and NDP52 were detected in whole cell protein extracts from peripheral lung tissue obtained from COPD patients and age-matched healthy subjects and smokers with normal lung function (Table 1). The levels of galectin-8 and NDP52 were significantly higher in COPD compared with controls with normal lung function (Fig. 5a,b and supplementary Fig. S9).

**Table 1:** COPD study patient characteristics for lung study.

	Healthy volunteers	Smokers with normal lung function	COPD GOLD1	COPD GOLD2	COPD GOLD3	COPD GOLD4
N	4	11	8	7	4	5
Male/female	2/2	3/8	5/3	4/3	3/1	2/3
Age (years)	56.0 ± 26.5	63.6 ± 12.2	68.5 ± 6.8	60.4 ± 8.7	64.0 ± 8.4	60.2 ± 3.2
FEV_1_ (L)	2.75 ± 1.26	2.57 ± 0.64	2.65 ± 0.56	1.81 ± 0.39	1.63 ± 0.47	0.48 ± 0.09*
FEV_1_ (%predicted)	89.6 ± 4.8	98.9 ± 11.9	91.4 ± 7.54	58.4 ± 8.3**	46.7 ± 1.3*	16.3 ± 2.2*
FEV_1_/FVC (%)	79.6 ± 5.5	74.6 ± 4.0	63.3 ± 4.4**	59.6 ± 9.9**	51.5 ± 8.3*	24.2 ± 3.0*

Data are expressed as mean values ± SD. *P < 0.05; **P < 0.01 versus healthy volunteers.

Furthermore, the level of secreted galectin-8 was determined in serum obtained from 31 healthy volunteers and 26 stable COPD patients (Table 2). There were no correlations between galectin-8 and lung function and smoking history (Table 3), but it was observed that COPD patients who suffered from frequent acute exacerbations (2 or more/year) had significantly higher levels of galectin-8 in their serum (383.8 ± 248.3 pg/ml) compared to healthy volunteers (209.1 ± 120.8 pg/ml), and also when compared to COPD patients with infrequent exacerbations (176.2 ± 100.0 pg/ml) (Fig. 5c). Furthermore, increased serum galectin-8 was also correlated with reduced 6-min walking distance (6MWD) in COPD patients (Fig. 5d).

**Table 2:** COPD patient characteristics for serum study.

	HV + SM	COPD		Stats
		Infrequent exacerbator	Frequent exacerbator	
n	31	16	10	
Male	45%	88%	70%	*P* < 0.05
Age (y.o.)	61.4 ± 12.1	61.0 ± 9.6	69.8 ± 9.6	NS
Smoker	61%	100%	100%	*P* < 0.01
Pack-years	23.8 ± 27.5	65.1 ± 26.3	76.5 ± 25.4	*P* < 0.0001
MRC dyspnea score	0.55 ± 0.93	1.56 ± 1.03	2.50 ± 0.85	*P* < 0.0001
Body mass index (kg/m^2^)	27.7 ± 6.3	24.0 ± 3.2	23.7 ± 4.3	*P* < 0.05
FEV_1_ / FVC (%)	82.8 ± 8.0	57.1 ± 10.8	48.1 ± 8.7	*P* < 0.0001
FEV_1_ (% predicted)	89.6 ± 13.5	57.5 ± 24.7	44.9 ± 21.1	*P* < 0.0001
FVC (% predicted)	86.1 ± 13.5	74.3 ± 19.2	72.8 ± 28.8	*P* < 0.05
6MWD (m)	497.6 ± 108.2	451.7 ± 129.7	315.8 ± 113.5	*P* < 0.001

Data are expressed as mean values ± SD. Frequent exacerbator is defined as patients having two or more exacerbations in 1 year.

*HV* healthy volunteers, *SM* smokers, *MRC* Medical Research Council, *6MWD* six minute walk distance.

**Table 3:** Correlation of lung and serum galectin-8 with clinical parameter.

	Lung				Serum			
	All	COPD only	COPD (GOLD1 + 2)	COPD (GOLD3 + 4)	All	COPD only	COPD (GOLD1 + 2)	COPD (GOLD3 + 4)
Age	0.2593	0.6442	0.8951	0.1536	0.0718	0.1447	0.2212	0.5199
Pack-year	0.6373	0.3741	0.4166	0.8063	0.2563	0.3311	0.3227	0.9522
MRC dyspnea score	N/A	N/A	N/A	N/A	0.1212	0.2680	0.0450	0.9881
Body mass index (kg/m^2^)	N/A	N/A	N/A	N/A	0.7556	0.8879	0.7370	0.5786
FEV_1_ / FVC (%)	0.0671	0.5086	0.8150	0.5887	0.1795	0.1011	0.0251	0.1198
FEV_1_ (% predicted)	0.0805	0.4198	0.6763	0.3401	0.2737	0.8696	0.7097	0.8491
FVC (% predicted)	N/A	N/A	N/A	N/A	0.3937	0.4017	0.9166	0.4115
6MWD (m)	N/A	N/A	N/A	N/A	0.0030	0.0125	0.1435	0.1706

Data are *P* values of correlation coefficient.

*N/A* not available, *MRC* Medical Research Council, *6MWD* six minute walk distance, *GOLD* Global Initiative for Chronic Obstructive Lung Disease.

#### Discussion

Recently the link between cigarette smoking and defective autophagy has been strengthened, with several studies providing evidence for altered cell survival and increased mucus production^10,11,26^. Most previous studies, including our own, have focused on the interaction between CSE and the airway epithelium^13^, however we have now shown that CSE exposure also causes accumulation of the autophagy-associated protein LC3, and aggregation of p62 within macrophage-like cells. p62 acts as an adaptor protein that recognises ubiquitinated proteins and transfers them to the autophagosome for degradation^27^. Accumulation of high molecular weight (HMW) p62 within a cell is a sign that autophagosomes are not maturing into autolysosomes and may be a marker of defective autophagic flux^14^. The presence of HMW-p62 has been reported to be associated with reactive oxygen species (ROS) and can be induced by CSE and UV exposure^14,28^. Using three independent methodologies (Fig. 1d,f,g) we have shown that CSE inhibits autophagic flux leading to defective autophagosome maturation and accumulation of autophagosome cargo within macrophages. These data support previously reported observations in alveolar macrophages from smokers^14^ and are consistent with our previous data obtained using bronchial epithelial cells which are a primary target of cigarette smoke exposure^13^. Our results strengthen the mechanistic understanding of autophagosome accumulation in COPD.

Galectin-8 is an autophagy adaptor protein and works in concert with NDP52 to activate autophagy-mediated clearance of bacteria^15,29^. Galectin-8 has been reported to monitor endosomal and lysosomal integrity, and serves as a receptor for damaged vesicles, initiating selective autophagic degradation via the binding of NDP52^15,17^. Our experiments demonstrated that both galectin-8 and NDP52 undergo constitutive degradation by autophagic clearance mechanisms, with both proteins accumulating intracellularly in the presence of the autophagosome-lysosome fusion inhibitor bafilomycin A (Fig. 2a). Exposure of macrophages to CSE led to accumulation of intracellular galectin-8 and NDP52, confirming a role for cigarette smoke in the raised levels of these proteins seen in COPD patients, and supporting the supposition that cigarette smoke inhibits autophagosome maturation.

As a result of its function in the determination of endosomal and lysosomal vesicle integrity, galectin-8 serves as a danger receptor for vesicle-damaging bacteria, leading to selective autophagic degradation of the bacteria through its interaction with NDP52^15^. COPD is associated with chronic bacterial colonisation of the lower airways, which is associated with disease severity^30^. Bacterial colonisation is related to greater airway inflammation and frequent acute exacerbations^31,32^. Bacterial infection is a common trigger of acute exacerbations in COPD patients, accelerating lung function decline, worsening quality of life and increasing mortality^33^. Here we show that increased galectin-8 in the serum of COPD patients was associated with greater exacerbation frequency. Furthermore, secretion of galectin-8 was shown to be increased by CSE in macrophage-like cells. As galectin-8 is a secreted protein^34^, increased serum galectin-8 may simply reflect accumulation within cells due to defects in autophagic clearance mechanisms caused by cigarette smoke. However, an unconventional autophagy-based secretory pathway has recently been described for IL-1β, and therefore it is possible that galectin-8 is actively secreted from the cell by autophagic mechanisms^35^.

Systemic inflammation in COPD patients, particularly increased levels of the inflammatory cytokine IL-6, is associated with reduced FEV_1_ and 6MWD^36–38^. IL-6 initiates many pro-inflammatory processes and regulates the expression of C-reactive protein (CRP), a marker of inflammation in COPD^39^. Galectin-8 plays a role in the control of IL-6 expression in human endothelial cells (HMEC-1) and splenocytes from mice, and conversely to be mediated by IL-6 expression itself, suggesting a positive feedback mechanism^40,41^. We also observed galectin-8 dependent IL-6 release in bronchial epithelial cells (Fig. 4a,b, supplementary Fig. S7). Furthermore, in this study we have shown that patients with increased 6MWD express lower levels of serum galectin-8 suggesting a possible mechanistic relationship between galectin-8 expression in COPD patients and the IL-6-associated reduction in 6MWD.

The mechanism of action of secreted galectin-8 induced IL-6 release is poorly understood. It has previously been shown that immobilised galectin-8 activates Ras leading to increased phosphorylation of ERK1 and ERK2, while addition of the pan-phosphoinositide 3-kinase (PI3K) inhibitor wortmannin nullifies this effect^42^. The role of PI3K in lung disease is well established^43^. In the current study, galectin-8 dependent IL-6 induction in bronchial epithelial cells was shown to be significantly reduced by inhibiting PI3Kα, the predominant isoform in bronchial epithelial cells, rather than PI3Kγ and PI3Kδ. In addition, p38 MAPK α/β signalling also be involved in IL-6 production in response to galectin-8 as p38 MAPK inhibitor SB203580 partially reduced IL-6 secretion.

Non-apoptotic cell death has recently attracted attention as a contributing factor to the pathogenesis of COPD^44^. In contrast to apoptosis, non-apoptotic cell death, such as necroptosis and ferroptosis, is accompanied by a rupture of plasma membrane resulting in a release of damage-associated molecular patterns (DAMPs) from dying cells, which are strong inducers of inflammation^45^. Markers of necroptosis are increased in COPD lung^46^. In addition, cigarette smoke exposure induces necroptosis and the subsequent release of DAMPs causes airway inflammation in mice^47^. Ferroptosis is also identified in airway epithelial cells exposed to cigarette smoke and in COPD lung^48^. We observed that galectin-8 induced non-apoptotic cell death. Although the mechanism underlying non-apoptotic cell death caused by galectin-8 is unknown, galectin-8 may contribute the pathogenesis of COPD via the induction of non-apoptotic cell death.

Accumulation of dysfunctional alveolar macrophages within the lung is a characteristic of COPD progression and associated with an inflammatory microenvironment^49,50^. As CSE inhibits autophagosome maturation and degradation in macrophage like cells, and consequent intracellular and extracellular concentrations of galectin-8 are altered leading to the activation of pro-inflammatory signalling cascades, the galectin-8/NDP52 pathway may prove to be a novel therapeutic target in COPD.

## Methods

### Reagents

Antibodies against the following were used for immunoblotting: NDP52 (Novus Biologicals, Abcam), LC3 (Cell Signaling Technology), p62 (Sigma), Galectin-8 (R&D Systems, Abcam), β-actin (Abcam), anti-mouse (Dako) and anti-rabbit (Dako). For inhibition of PI3K isoforms or p38 MAPK the following compounds were used: PIK75 (Abcam), AS-605240 (VWR), IC-87114 (VWR) and SB203580 (Sigma).

### Cell culture

U937 cells (ATCC, LGC Standards) were cultured at 37 °C and 5% CO_2_ in RPMI-1640 supplemented with 10% heat-inactivated foetal bovine serum (FBS) and 15 mM L-glutamine. When required, U937 cells were differentiated into a macrophage-like phenotype by exposure to 50 ng/ml of PMA for 72 h, as described previously^51^. BEAS-2B cells (ATCC, LGC Standards) were cultured in keratinocyte basal medium (KBM) supplemented with human epidermal growth factor, insulin, hydrocortisone, calcium, bovine pituitary extract, and gentamicin sulphate amphotericin-B (GA-1000) (Lonza). PMA-differentiated U937 macrophage-like cells were serum starved 16 h before stimulation using RPMI-1640 supplemented with 1% FBS and 15 mM L-glutamine, whilst BEAS2B cells were serum starved with non-supplemented keratinocyte media.

### Patients

Disease severity in COPD patients was graded following the Global Initiative for Obstructive Lung Disease (GOLD) severity score^52^. In cohort 1 we obtained lung tissue from a tissue bank linked to an established patient registry^53^. Specimens of peripheral lung tissue were obtained from 4 healthy volunteers, 11 smokers without symptoms, 8 subjects with GOLD 1 COPD (mild), 7 with GOLD 2 COPD (moderate), 4 with GOLD 3 COPD (severe) and 5 with GOLD 4 COPD (very severe). In cohort 2 serum samples were obtained from 31 healthy volunteers and 26 stable COPD patients (GOLD 1 n = 5, GOLD 2 n = 8, GOLD 3 n = 8, GOLD 4 n = 5). We excluded patients with cancer and patients who had episodes of acute exacerbations or respiratory tract infections in the last eight weeks. Frequent exacerbations were defined as two or more episodes in the last year.

Patient samples were obtained with approval from the ethics committee of the Royal Brompton and Harefield Hospitals National Health Service Trust and the Sismanogleio General Hospital (Approval number is 5210-07/03/2012) and all subjects provided written informed consent. This study was performed in accordance with the ethical standards as laid down in the 1964 Declaration of Helsinki and its later amendments or comparable ethical standards.

### Preparation of cigarette smoke extract

CSE was prepared as described previously^54^. Briefly, the smoke from one full-strength Marlboro cigarette with filter removed (Phillip Morris) was passed through 10 ml of RPMI-1640 supplemented with 1% FCS and 15 mM L-glutamine, using a peristaltic pump. CSE was filter sterilised by passing the solution through a 0.2 μm filter. The optical density was measured at OD_320_ wavelength, and values were diluted to achieve a value of 0.85 ± 0.03, which was considered 100% CSE.

### Preparation of cell extracts and western blotting

Extraction of protein from lung tissue was performed as described previously^55^. Whole cell extraction of protein from cell lines was performed as described previously^56^. Cell lysates were analysed by SDS-PAGE (Invitrogen) and specific proteins were detected using antibody probes followed by quantification of chemiluminescence (ECL Plus, GE Healthcare).

### MTT assay

Cells were washed with PBS and 100 μl of medium containing MTT (final concentration 0.5 mg/ml) was added. After 2 h incubation at 37 °C and 5% CO_2_, MTT solution was aspirated and 100 μl DMSO was added. Following 30 min incubation at room temperature absorbance was measured at OD_570_.

### Overexpression of GFP-LC3 and confocal microscopy

GFP-LC3 plasmid was purchased from Addgene and transfected into PMA-differentiated U937 macrophage-like cells cultivated on collagen-coated coverslips using Attractene transfection reagent (Qiagen). Following transfection, cells were rinsed with 0.05% Saponin in PBS and then fixed with 4% paraformaldehyde in PBS for 20 min. After washing the cells with PBS twice, the coverslips were washed with distilled water once and then mounted onto glass slides. Images were taken with a 63 × Plan Apochromat 1.4 NA objective on a Zeiss LSM-510 inverted confocal microscope (Carl Zeiss, Jena, Germany) at room temperature. Images were analysed and processed using ZEN acquisition software. To quantify the percentage of cells with GFP-LC3 puncta, at least 50 cells per experiment were counted in randomly selected fields.

### Real-time quantitative PCR

Total cellular RNA was extracted using the RNeasy kit (Qiagen) and cDNA was prepared using the High capacity cDNA reverse transcription kit (Applied Biosystems). Real-time quantitative PCR (RT-qPCR) analyses of NDP52, Galectin-8 and GNB2L1 were performed using TaqMan primers and probe sets from Thermo Fisher Scientific on the Applied Biosystems 7500 Real-Time PCR System.

### ELISA

To determine expression of secreted proteins ELISAs were performed according to the manufacturer’s guidelines. Galectin-8 released into supernatant was measured by ELISA (RayBiotech) and if necessary results were normalised using the MTT assay. Serum Galectin-8 was measured using a high sensitivity ELISA (Cusabio). IL-6 in supernatant was measured by ELISA (R&D Systems, Minneapolis, MN, USA) or by electrochemiluminescence (Meso Scale Discovery).

### Knock-down of NDP52 expression

PMA-differentiated U937 cells were transfected with NDP52 siRNA (Cell Signaling Technology) or negative control siRNA (Thermo Fisher Scientific) in OPTI-MEM using Lipofectamine RNAiMAX (Thermo Fisher Scientific). After 48 h incubation at 37˚C and 5% CO_2_ transfected cells were ready for use.

### Immunoprecipitation

PMA-differentiated U937 cells were collected and lysed by RIPA buffer. Then the protein was incubated with NDP52 antibody (Abcam) or rabbit IgG isotype control antibody (Proteintech) at 4 °C overnight, and then incubated with Pierce Protein A/G Magnetic Beads (Thermo scientific) at room temperature for 1 h. The immune complex was eluted at 100 °C for 5 min. The eluate was subjected to SDS-PAGE for Western blotting.

### Detection of apoptosis

Apoptosis was evaluated by fluorescent microscope and flowcytometry, using an Apoptosis/Necrosis detection kit (Abcam) and Pacific Blue Annexin V Apoptosis Detection Kit (Biolegend), respectively. After appropriate treatments, cells were processed according to the manufacturer’s instructions. In microscopy experiments, a minimum of 1000 cells were counted per condition per experiment using Fiji software (http://fiji.sc/Fiji). The data of flowcytometry were analysed with FlowJo software.

### Caspase 3/7 activity assay

The activities of caspase-3/7 activity were evaluated using an Apo-ONE Homogeneous Caspase-3/7 Assay Kit (Promega), according to the manufacturer’s protocols. Briefly, BEAS-2B cells were seeded into 96-well plates and incubated overnight, and then cells were treated with 500 nM of galectin-8 for 24 h. After that, Apo-ONE Caspase-3/7 Reagent was added to each well. Cells were incubated at room temperature for 1 h and the levels of luminescence of each well were measured. Caspase activities were determined by calculating the ratio of levels of luminescence of the treated cells to that of the non-treated cells, which was defined as 100%.

### LC3 turnover assay

PMA-differentiated U937 macrophage-like cells were cultured in the presence and absence of 30% CSE for 24 h. Subsequently, cells were untreated or exposed to 100 nM bafilomycin A for 2 h. Cells were harvested, whole cell proteins were extracted as described above and protein lysates were analysed by Western blotting. The expression of LC3-II was quantified and normalised against the house keeping protein β-actin.

### Long-lived protein degradation assay

Long-lived protein degradation was determined by the modified method described previously^57^. Briefly, PMA-differentiated U937 macrophage-like cells were cultured with methionine-free media containing homopropargylglycine (HPG) for 16 h to incorporate HPG into whole proteins. Then the cells were cultured with normal media for 2 h to remove HPG in short-lived proteins. After that, the cells were treated with CSE in the presence and absence of 10 nM bafilomycin A for 24 h. Finally, the amount of HPG was measured with FACS using a Click-it HPG Alexa Fluor Protein Synthesis kit (Thermo Fisher, Yorkshire, UK) according to the manufacturer’s instructions.

### Statistical analysis

Data are expressed as means ± SEM. Results were analysed using Mann–Whitney’s *U* test, Wilcoxon matched-pairs signed rank test, ordinary one-way ANOVA with Dunnett’s multiple comparisons test or RM ordinary one-way ANOVA with Dunnett’s multiple comparisons test, as appropriate. Correlation was analysed using spearman correlation test. Prism software (GraphPad Software, Inc., San Diego, CA) was used for statistical calculations. Experiments were repeated at least three times. *P* < 0.05 was considered statistically significant.

## Supplementary information

Supplementary Figures.

## Data Availability

The datasets generated during the current study are available from the corresponding author on reasonable request.
