# Supplementary material for: Cigarette smoke-induced impairment of autophagy in macrophages increases galectin-8 and inflammation
Source: Sci Rep. 2021 Jan 11;11:335. doi: 10.1038/s41598-020-79848-0 (PMC7801483; doi:10.1038/s41598-020-79848-0)
Supplement: Supplementary file 1 — Supplementary Figures. [file 41598_2020_79848_MOESM1_ESM.pdf]

Supplementary information

Cigarette smoke-induced impairment of autophagy in macrophages increases galectin-8 and inflammation

Yuta Kono[1, 2¶*], Thomas Colley[1¶], Masako To[3], Andriana I Papaioannou[4], Nicolas Mercado[1], Jonathan R Baker[1], Yasuo To[2], Shinji Abe[5], Kosuke Haruki[3], Kazuhiro Ito[1] and Peter J Barnes[1]

[1]National Heart and Lung Institute, Imperial College, London, London, United Kingdom.
[2]Deparment of Allergy and Respiratory Medicine, The Fraternity Memorial Hospital, Tokyo, Japan.
[3]Dapartment of Laboratory Medicine, Dokkyo Medical University Saitama Medical Centre, Saitama, Japan.
[4]3rd Respiratory Medicine Department, Sismanogleio Hospital, Marousi Athens Greece.
[5]Department of Respiratory Medicine, Tokyo Medical University Hospital, Tokyo, Japan.

¶ authors were equal contributors to this manuscript
* Corresponding author: Dr. Yuta Kono

Supplementary Figure S1

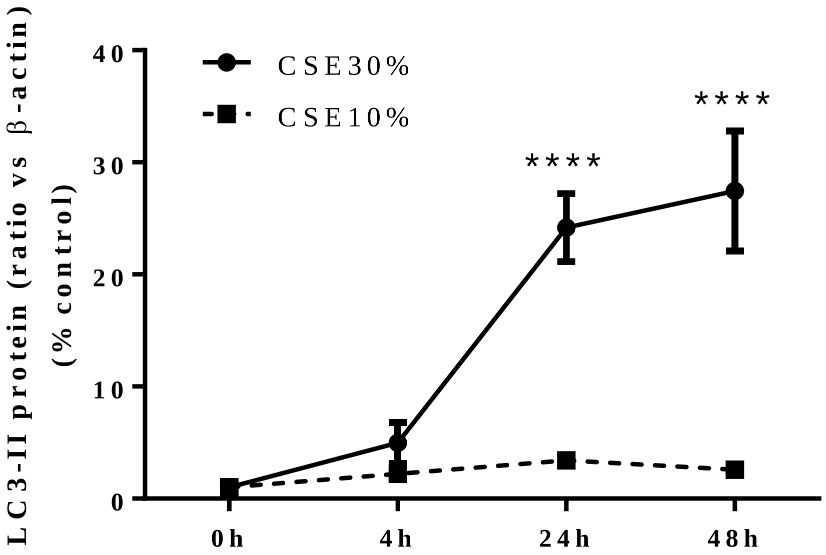

Supplementary Figure S1. Time and dose-dependency of CSE treatment on LC3-II expression. PMA-differentiated U937 macrophage-like cells were treated with 10%CSE and 30%CSE. Time course of LC3 expression was quantified by Western blot; n=3. **** $P < 0.0001$. CSE = cigarette smoke extract.

Supplementary Figure S2

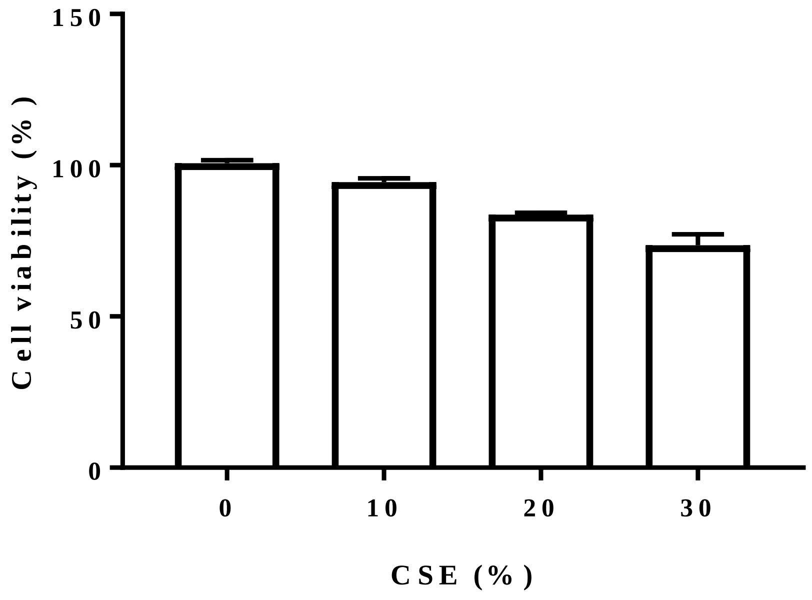

Supplementary Figure S2. Impact of CSE on PMA-differentiated U937 macrophage-like cell viability. Cell viability was quantified by MTT in PMA-differentiated U937 macrophage-like cells; n=3. CSE = cigarette smoke extract.

Supplementary Figure S3.

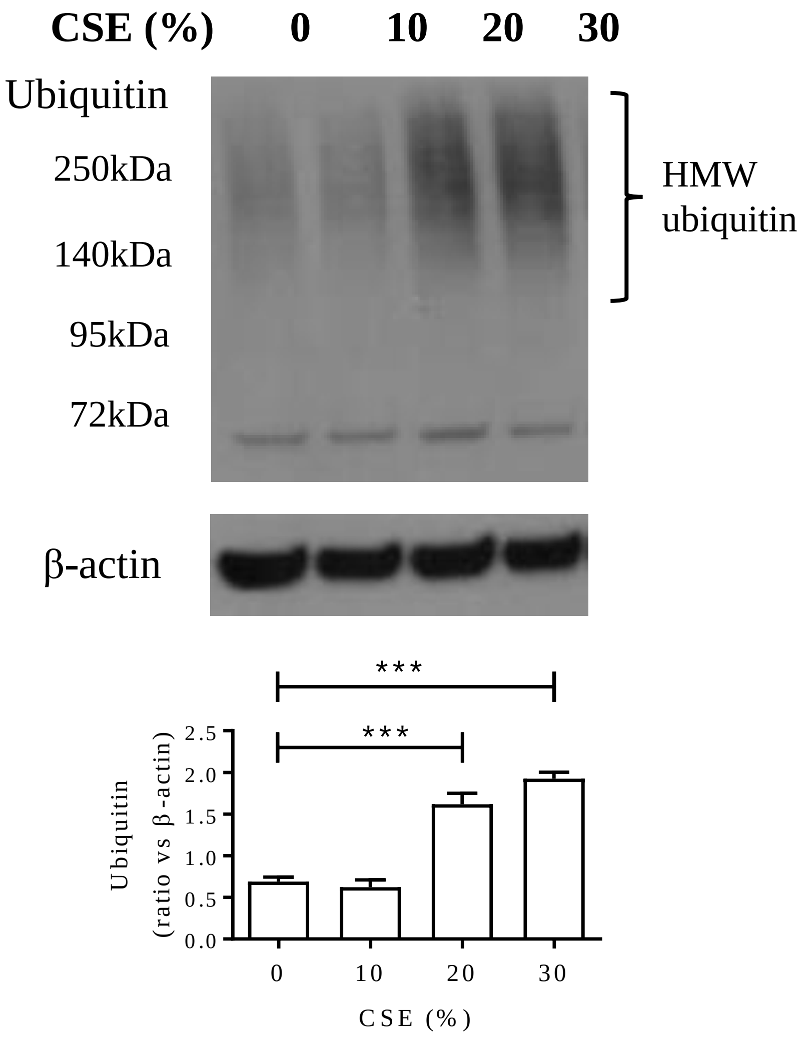

Supplementary Figure S3. HMW ubiquitin in PMA-differentiated U937 macrophage-like cells treated with CSE. PMA-differentiated U937 macrophage-like cells were treated with CSE and ubiquitin was quantified by Western blot; n=3. *** $P < 0.001$. CSE = cigarette smoke extract.

Supplementary Figure S4.

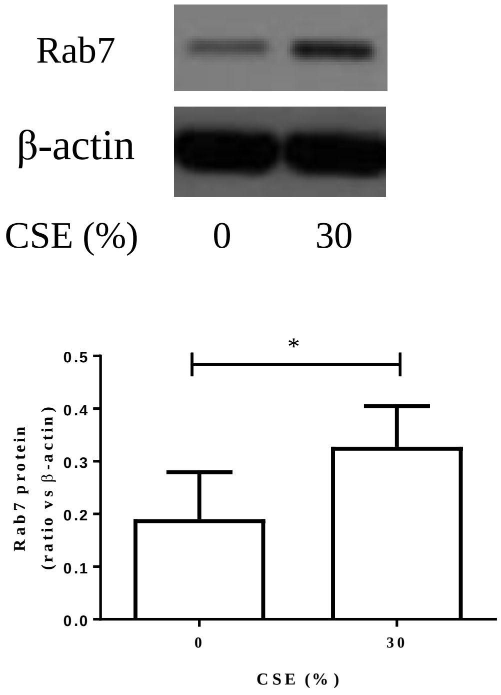

Supplementary Figure S4. Rab7 in PMA-differentiated U937 macrophage-like cells treated with CSE. PMA-differentiated U937 macrophage-like cells were treated with CSE and Rab7 expression was quantified by Western blot; n=3. * $P < 0.05$. CSE = cigarette smoke extract.

Supplementary Figure S5

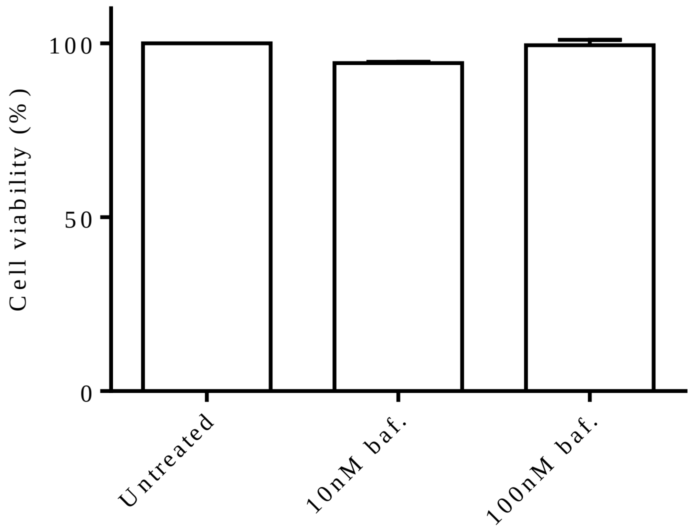

Supplementary Figure S5. Impact of bafilomycin A on PMA-differentiated U937 macrophage-like cell viability. Cell viability was quantified by MTT in PMA-differentiated U937 macrophage-like cells; n=3. CSE = cigarette smoke extract, baf = bafilomycin A.

Supplementary Figure S6

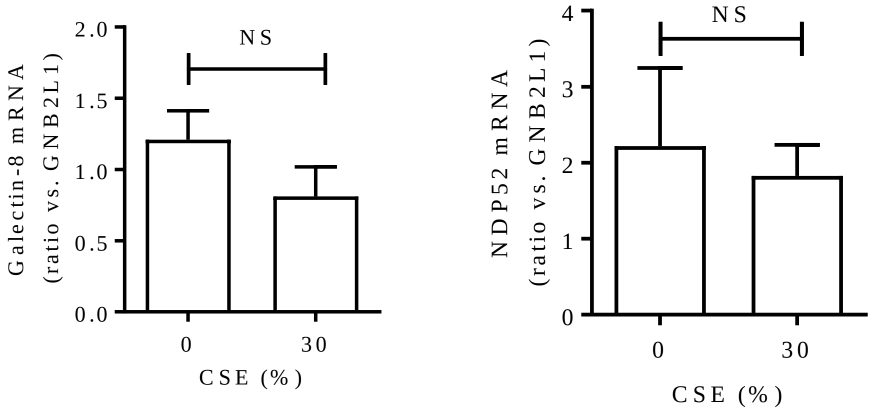

Supplementary Figure S6. Effects of CSE on mRNA transcription of Galactin-8 and NDP52. PMA-differentiated U937 macrophage-like cells were treated with CSE. mRNA transcription of Galectin-8 and NDP52 was quantified by Real-time quantitative PCR; n=3. CSE = cigarette smoke extract.

Supplementary Figure S7

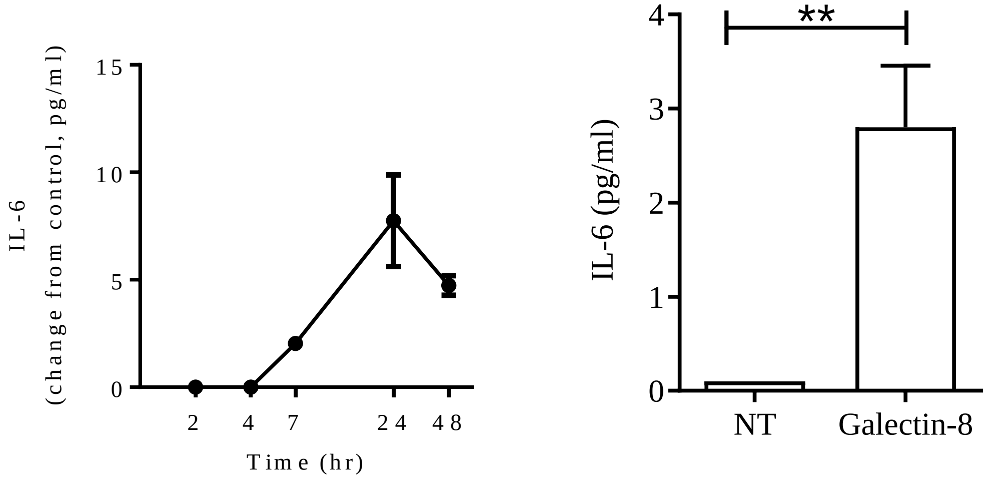

Supplementary Figure S7. Time course of galectin-8-induced IL-6 expression. IL-6 expression was quantified at 2, 4, 7, 24 and 48 h by ELISA in supernatant from BEAS-2B cells treated with 100 nM galectin-8, n=3. **Galectin-8-induced IL-6 expression in PMA-differentiated U937 macrophage-like cells.** IL-6 expression was quantified at 24 h by ELISA in supernatant from BEAS-2B cells treated with 100 nM galectin-8, n=3.

Supplementary Figure S8

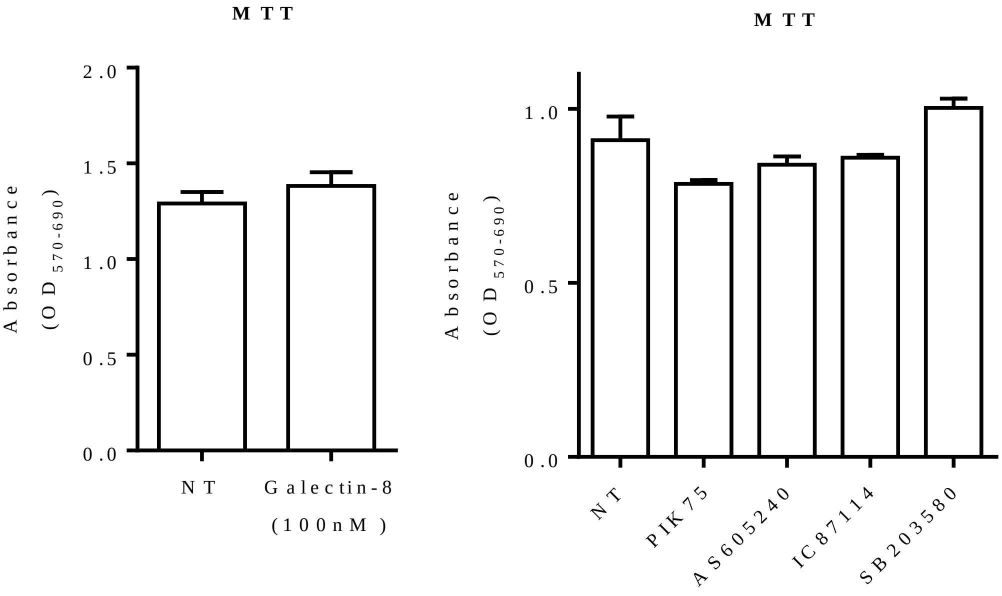

Supplementary Figure S8. Impact of galectin-8 and PI3K/p38 MAPK inhibitors on BEAS-2B cell viability. Cell viability was quantified by MTT in BEAS-2B cells; n=3.

Supplementary Figure S9

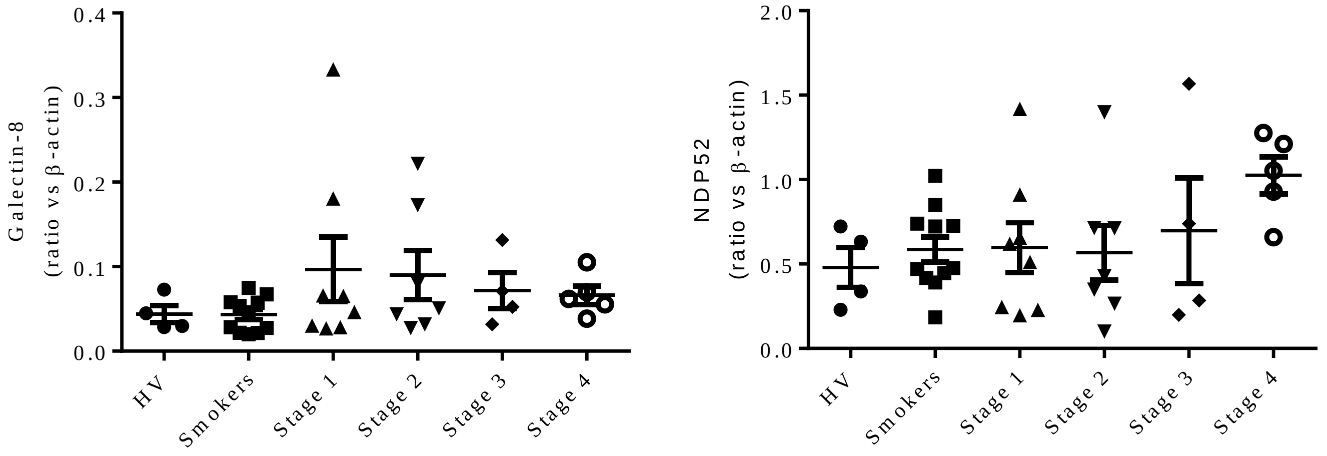

Supplementary Figure S9. Expression of galectin-8 and NDP52 in COPD, subdivided by disease stage. Galectin-8 and NDP52 protein in lung homogenates was quantified by western blot in healthy subjects (n = 4), smokers (n = 11), COPD GOLD stage 1 (n = 8), COPD GOLD stage 2 (n = 7), COPD GOLD stage 3 (n = 4) and COPD GOLD stage 4 (n = 5).

Supplementary Figure S10

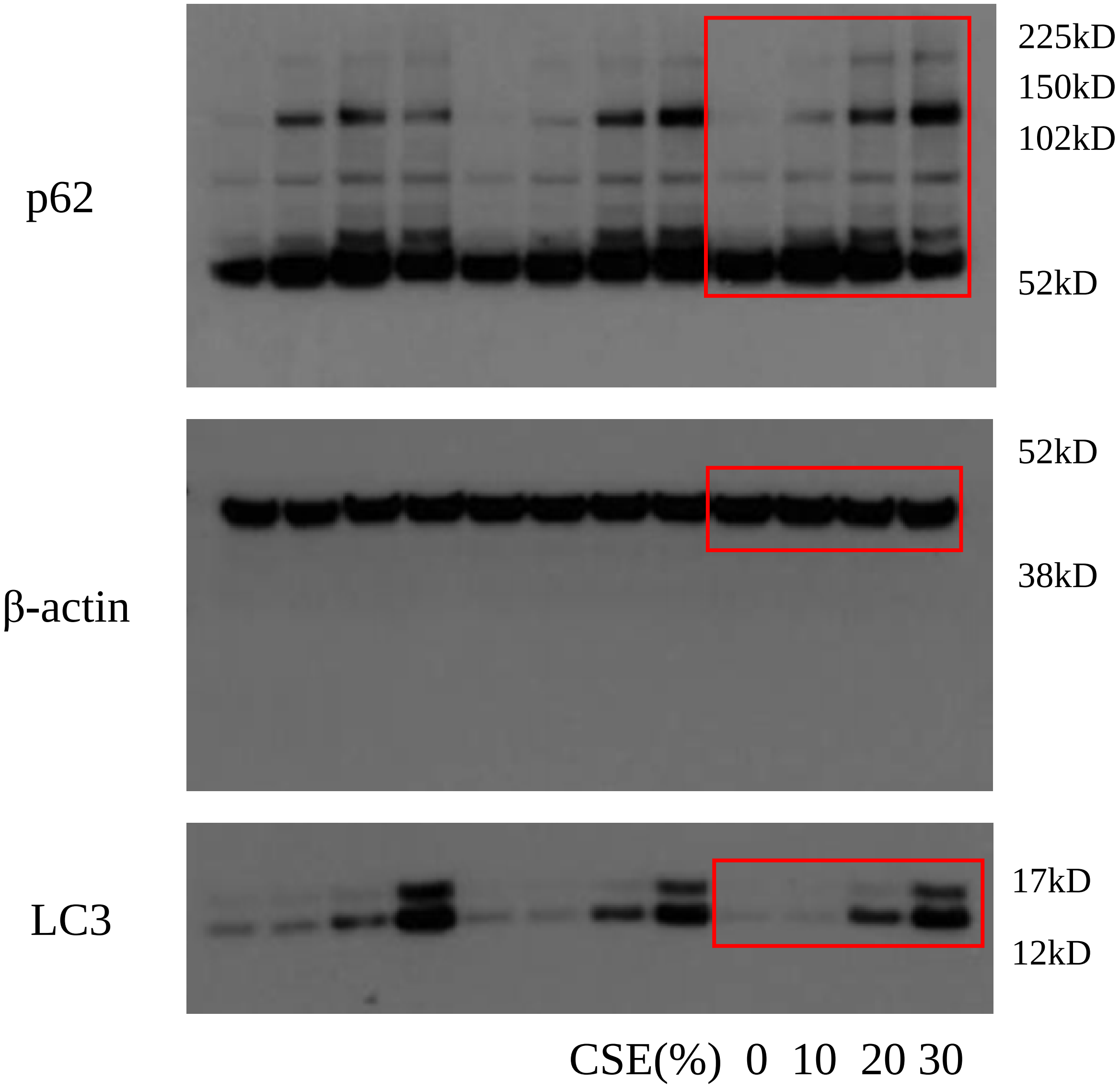

Supplementary Figure S10. Full-length Western blot images for Fig 1a.

Supplementary Figure S11

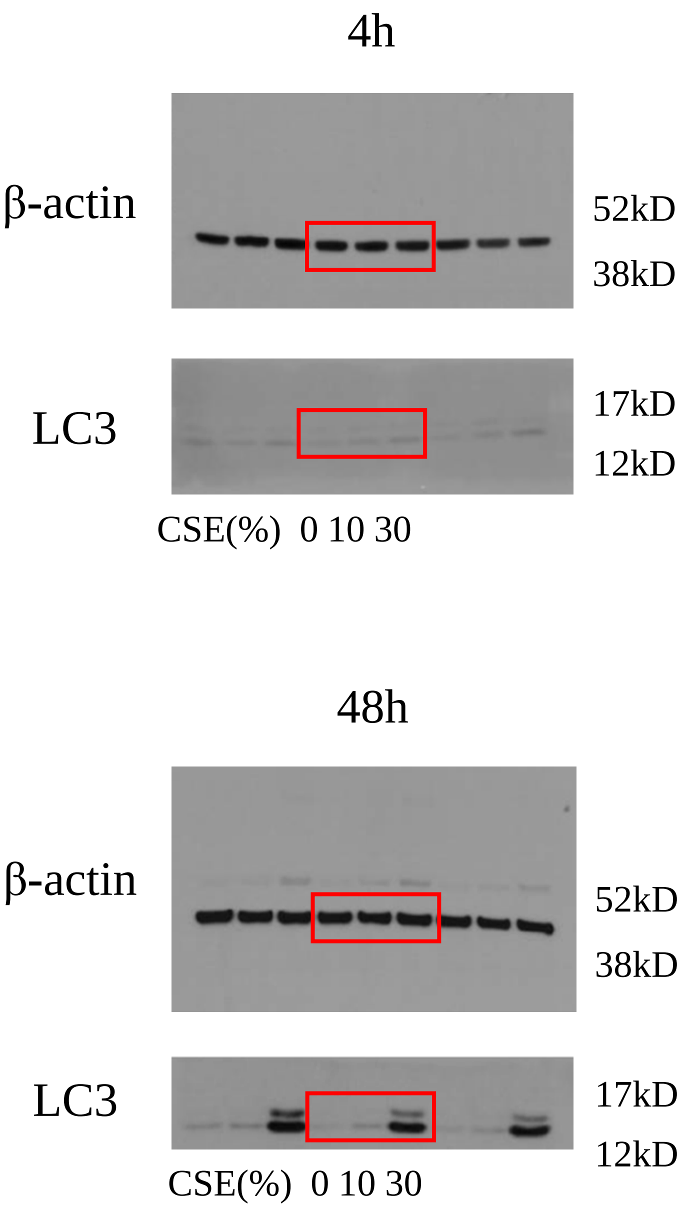

Supplementary Figure S11. Full-length Western blot images for Fig 1e.

Supplementary Figure S12

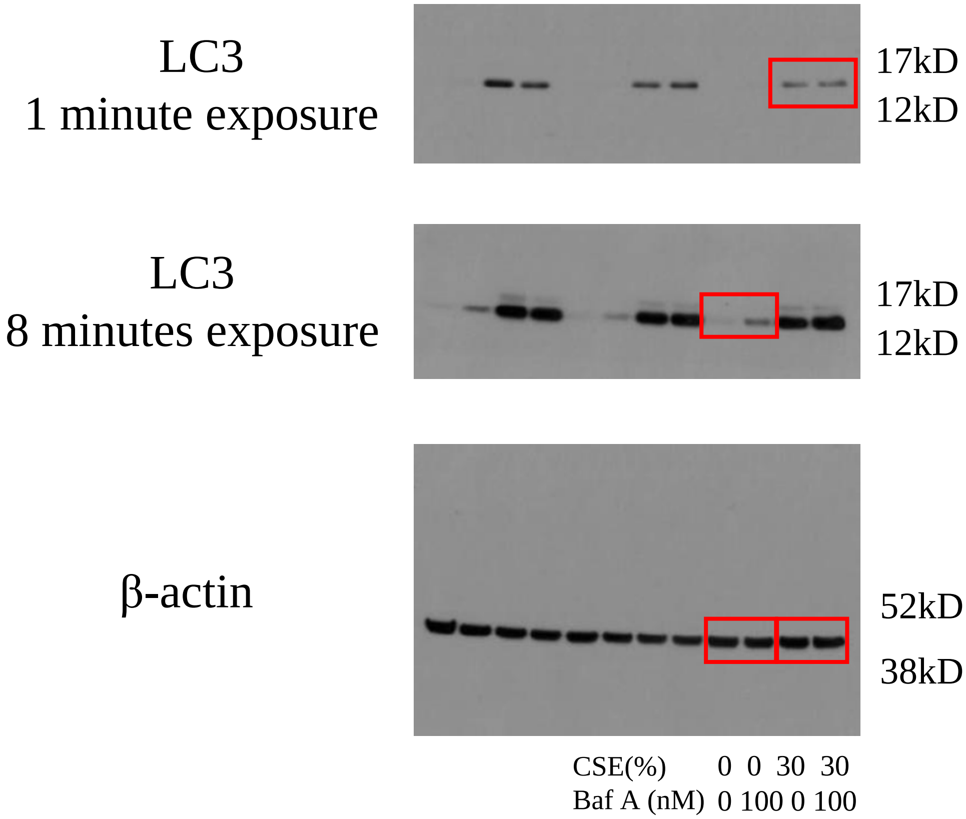

Supplementary Figure S12. Full-length Western blot images for Fig 1f.

Supplementary Figure S13

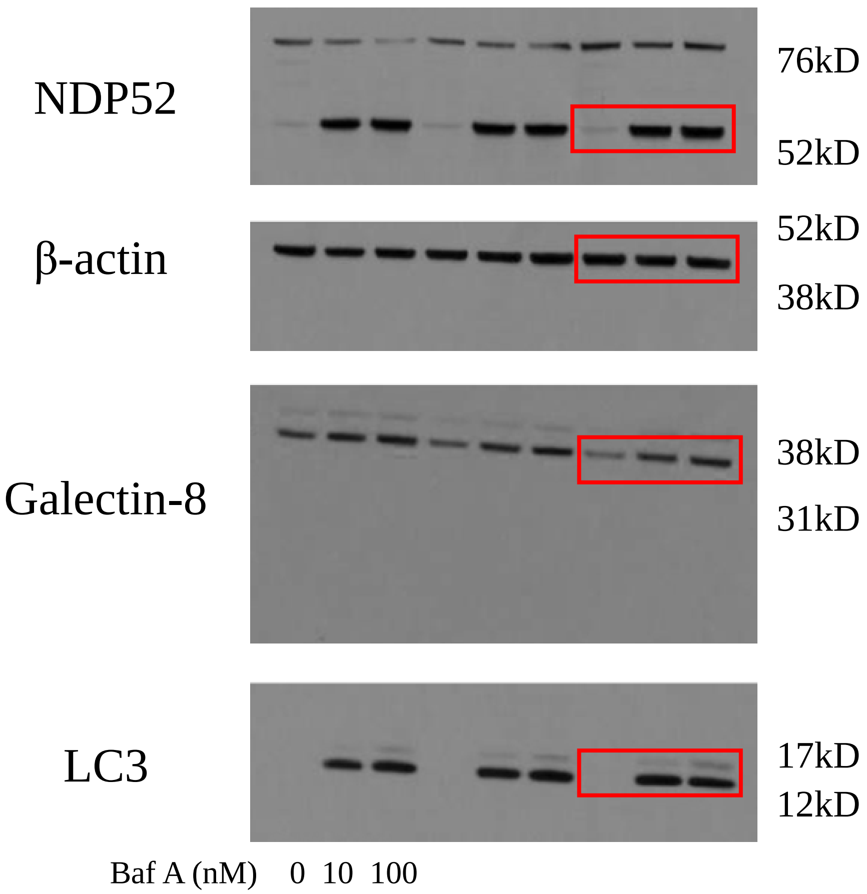

Supplementary Figure S13. Full-length Western blot images for Fig 2a.

Supplementary Figure S14

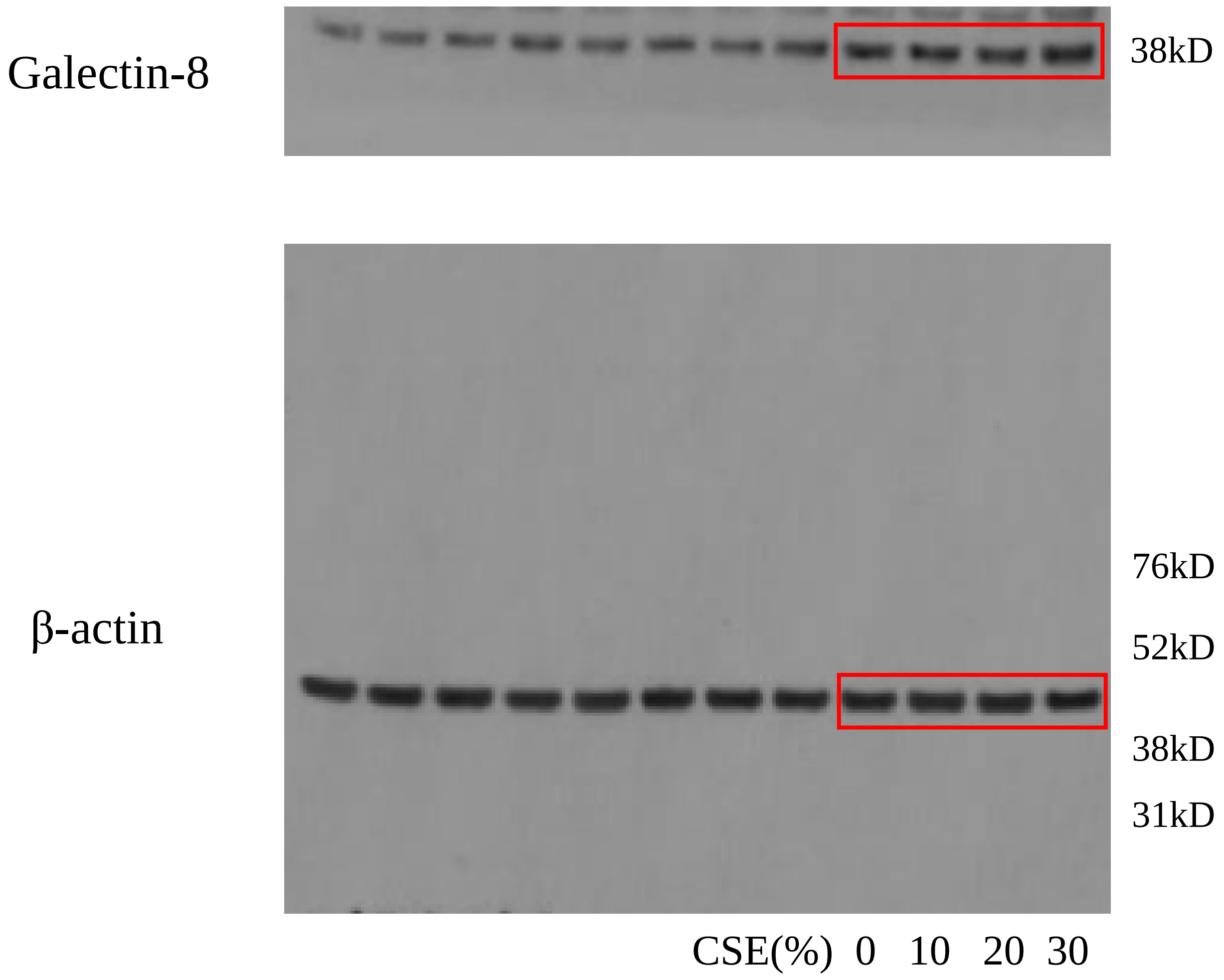

Supplementary Figure S14. Full-length Western blot images for Fig 2d.

Supplementary Figure S15

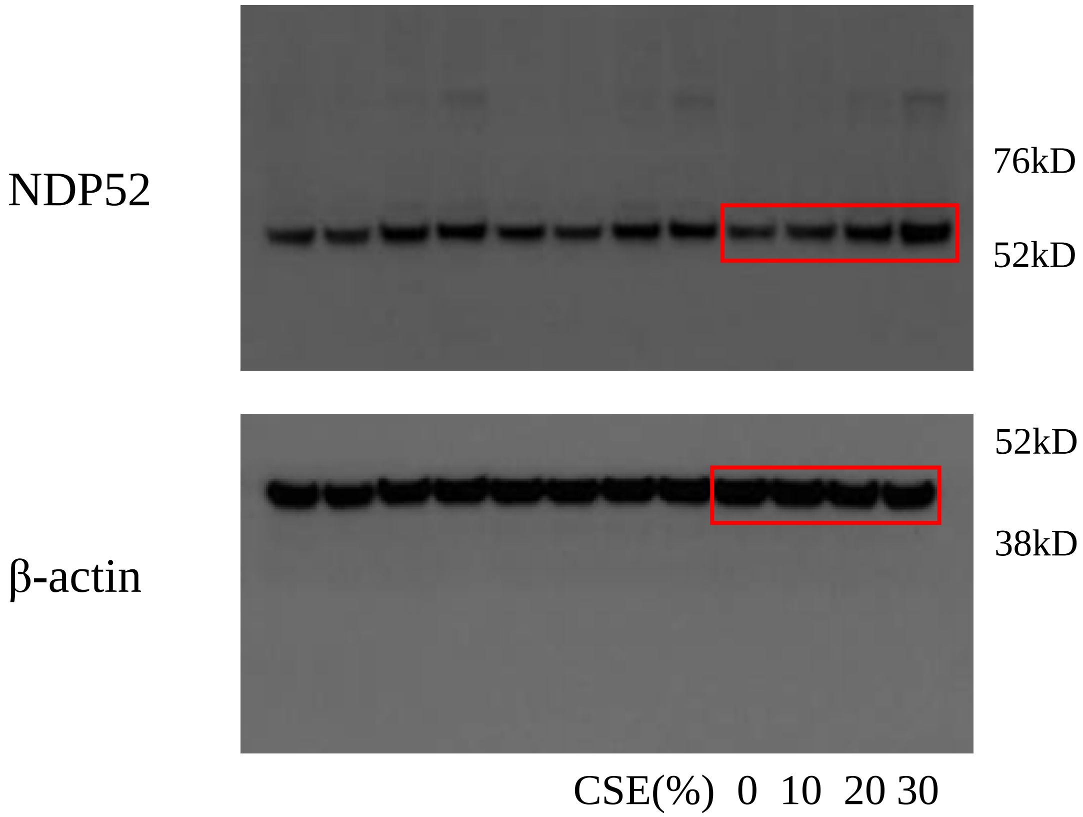

Supplementary Figure S15. Full-length Western blot images for Fig 2e.

Supplementary Figure S16

Galectin-8

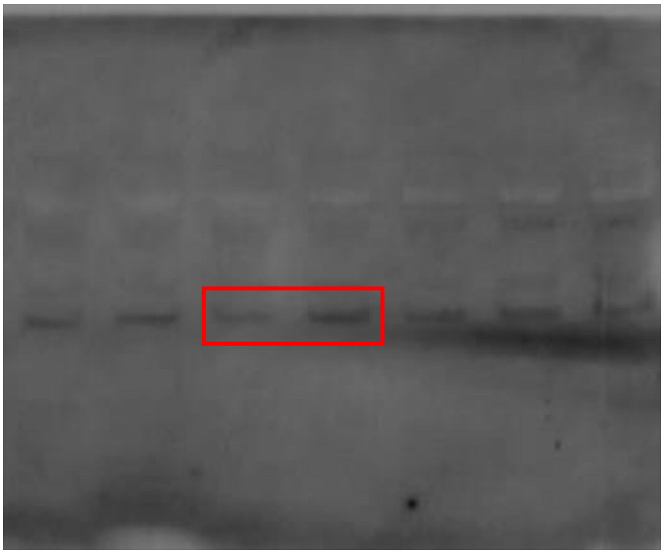

76kD
52kD
38kD
31kD

CSE(%) 0 30

Supplementary Figure S16. Full-length Western blot images for Fig 2f.

Supplementary Figure S17

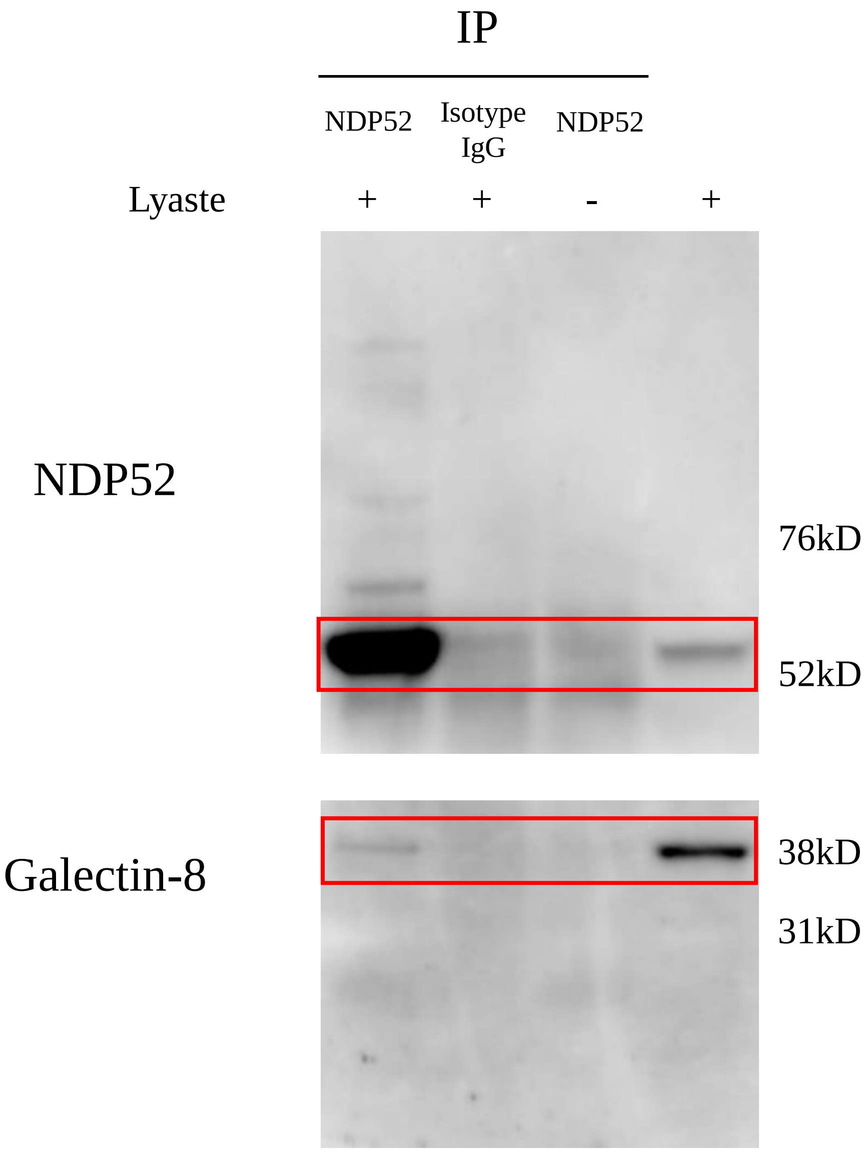

Supplementary Figure S17. Full-length Western blot images for Fig 3a.

Supplementary Figure S18

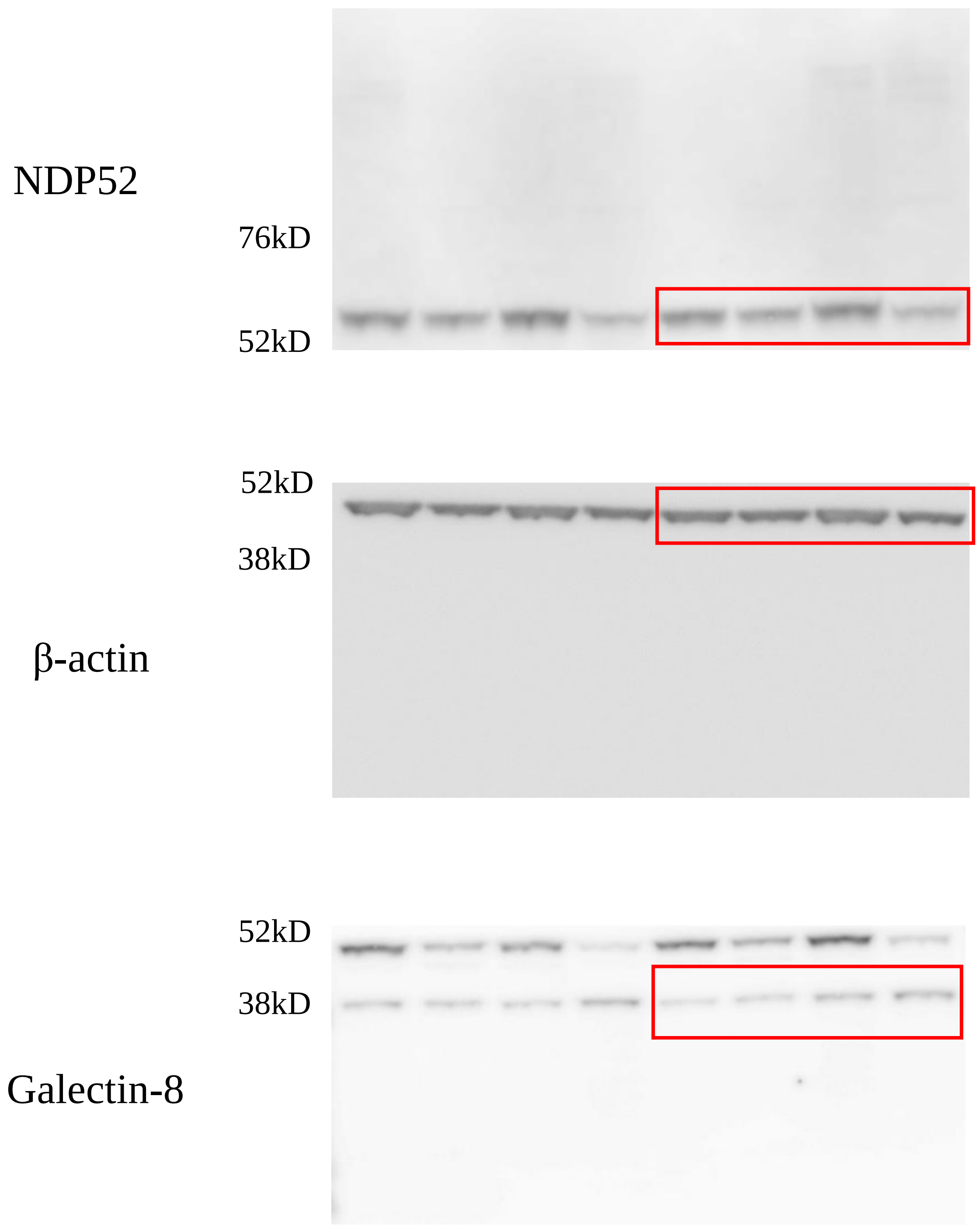

Supplementary Figure S18. Full-length Western blot images for Fig 3b.

Supplementary Figure S19

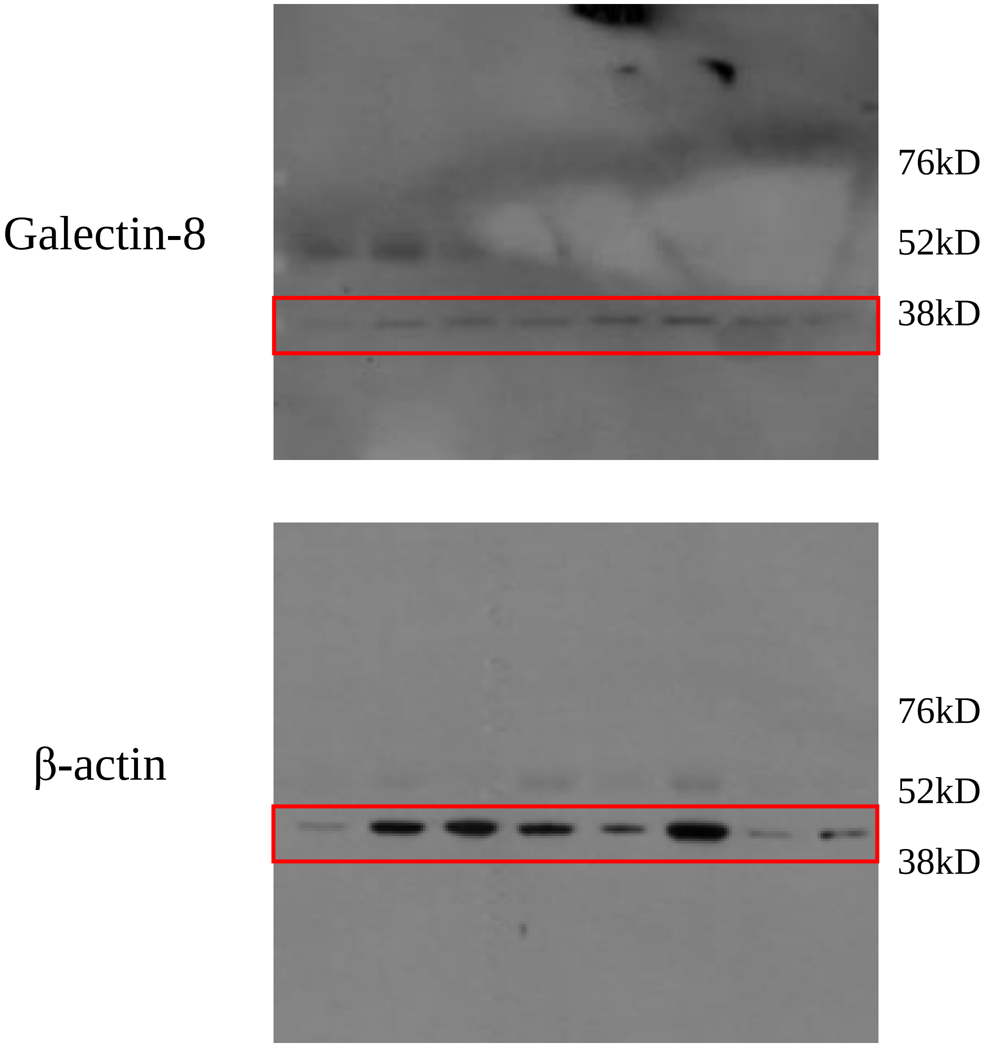

Supplementary Figure S19. Full-length Western blot images for Fig 5a.

Supplementary Figure S20

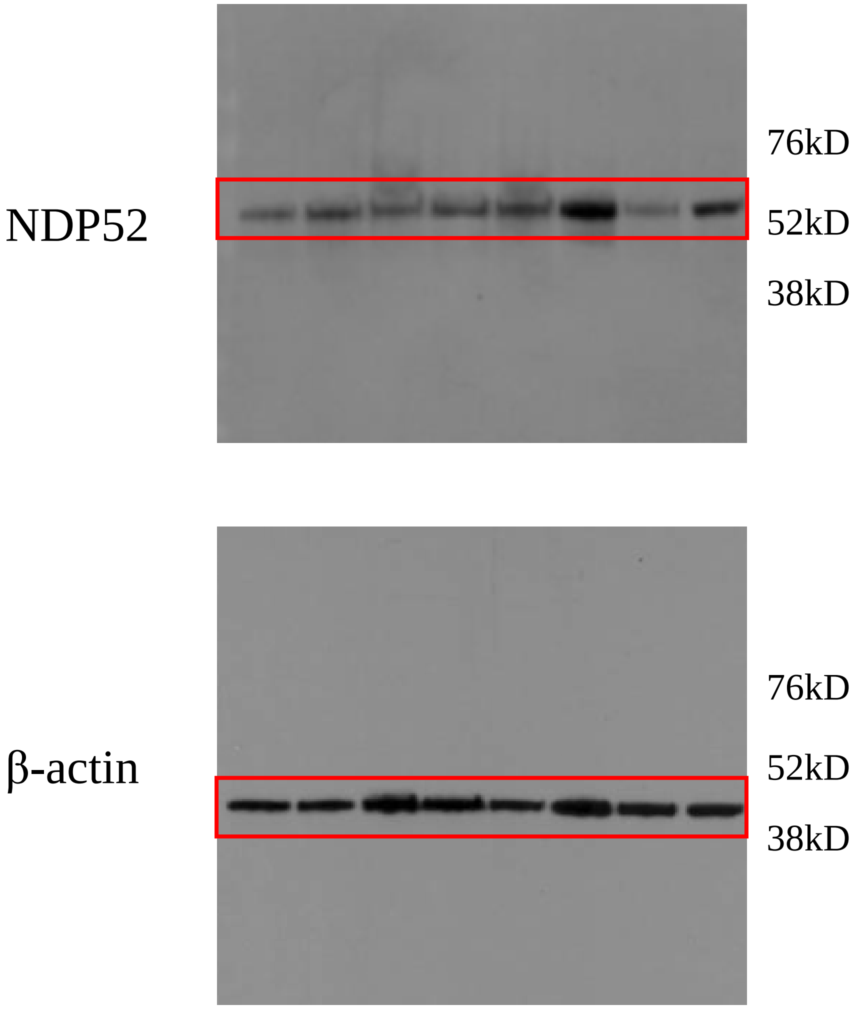

Supplementary Figure S20. Full-length Western blot images for Fig 5b.

Supplementary Figure S21

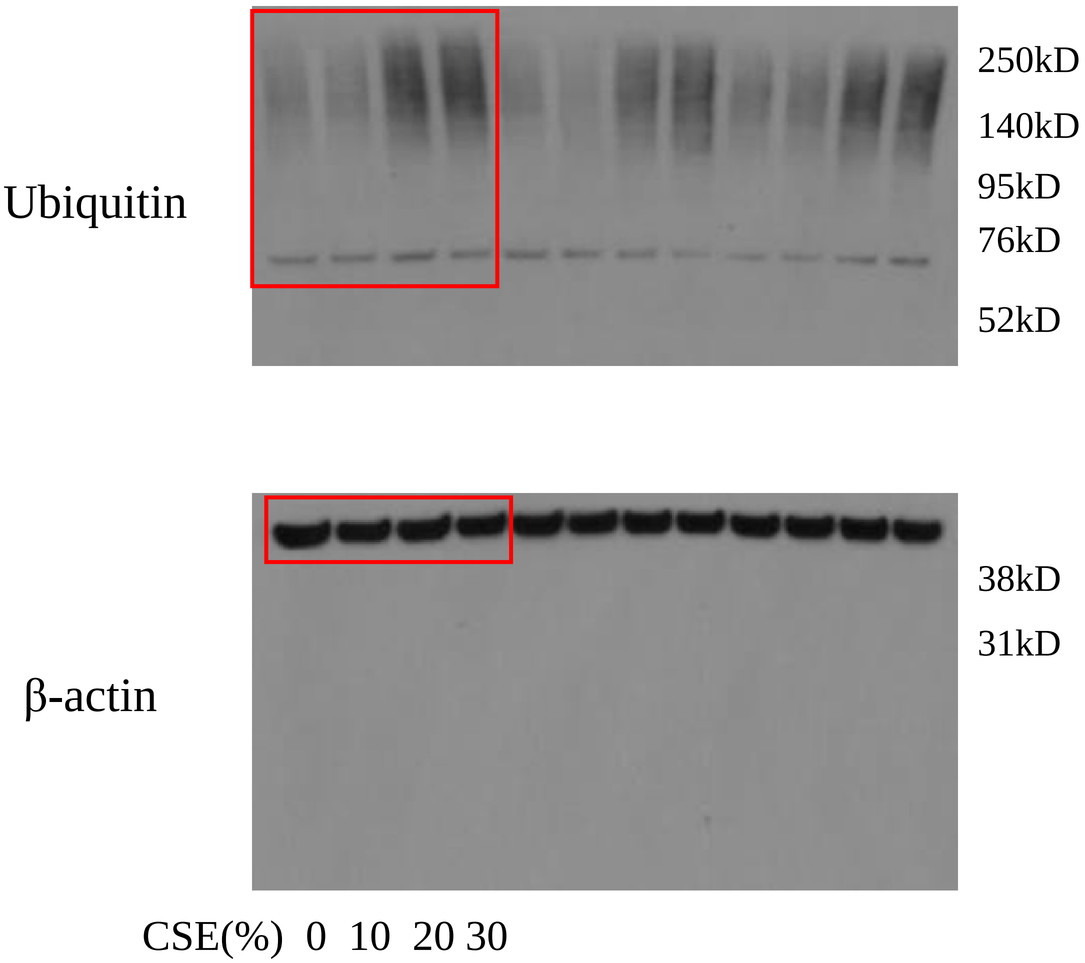

Supplementary Figure S21. Full-length Western blot images for Fig S3.

Supplementary Figure S22

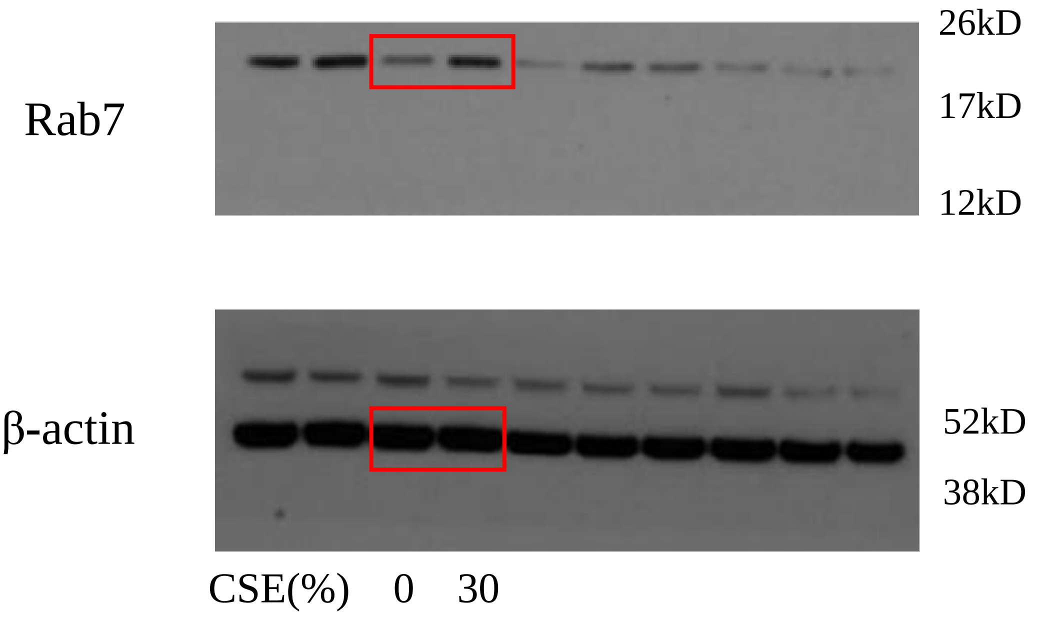

Supplementary Figure S22. Full-length Western blot images for Fig S4.
